# Differential modulation of the androgen receptor for prostate cancer therapy depends on the DNA response element

**DOI:** 10.1093/nar/gkaa178

**Published:** 2020-03-21

**Authors:** Steven Kregel, Pia Bagamasbad, Shihan He, Elizabeth LaPensee, Yemi Raji, Michele Brogley, Arul Chinnaiyan, Marcin Cieslik, Diane M Robins

**Affiliations:** 1 Michigan Center for Translational Pathology, University of Michigan, Ann Arbor, MI 48109, USA; 2 Department of Pathology, University of Michigan, Ann Arbor, MI 48109, USA; 3 Department of Human Genetics, University of Michigan, Ann Arbor, MI 48109, USA; 4 Department of Medicine and Urology, University of Michigan, Ann Arbor, MI 48109, USA; 5 Howard Hughes Medical Institute, University of Michigan, Ann Arbor, MI 48109, USA; 6 Rogel Cancer Center, University of Michigan, Ann Arbor, MI 48109, USA; 7 Bioinformatics, University of Michigan, Ann Arbor, MI 48109, USA

## Abstract

Androgen receptor (AR) action is a hallmark of prostate cancer (PCa) with androgen deprivation being standard therapy. Yet, resistance arises and aberrant AR signaling promotes disease. We sought compounds that inhibited genes driving cancer but not normal growth and hypothesized that genes with consensus androgen response elements (cAREs) drive proliferation but genes with selective elements (sAREs) promote differentiation. In a high-throughput promoter-dependent drug screen, doxorubicin (dox) exhibited this ability, acting on DNA rather than AR. This dox effect was observed at low doses for multiple AR target genes in multiple PCa cell lines and also occurred *in vivo*. Transcriptomic analyses revealed that low dox downregulated cell cycle genes while high dox upregulated DNA damage response genes. In chromatin immunoprecipitation (ChIP) assays with low dox, AR binding to sARE-containing enhancers increased, whereas AR was lost from cAREs. Further, ChIP-seq analysis revealed a subset of genes for which AR binding in low dox increased at pre-existing sites that included sites for prostate-specific factors such as FOXA1. AR dependence on cofactors at sAREs may be the basis for differential modulation by dox that preserves expression of genes for survival but not cancer progression. Repurposing of dox may provide unique opportunities for PCa treatment.

## INTRODUCTION

The progression of prostate cancer (PCa) depends on androgens acting via the androgen receptor (AR), and therefore androgen deprivation slows disease (1,2). However even with drugs that more effectively block androgen synthesis and AR transcriptional activity, resistance inevitably arises and AR regains control of disease (3,4). We proposed that selective modulation of AR to prevent expression of genes promoting cancer while retaining expression of genes for normal processes would slow progression, reduce selection pressure, and delay resistance. Existing pharmacological compounds that modulate steroid receptor function act via the ligand binding domain (LBD) to alter receptor interactions within transcription complexes, leading to gene-selective effects (5,6). Receptor domains other than the LBD are involved in selective gene expression through interactions with coregulators, chaperones, modifying enzymes, and the DNA binding site itself (7,8). Importantly, in late-stage PCa, AR variants lacking the LBD allow hormone-independent growth, highlighting the need to target other AR domains (9–11). Genomic data indicates that promoters and enhancers of genes related by function vary in response element sequence and in chromatin signatures (12–14), suggesting that shared variations such as these could also be targets for modulators.

The consensus androgen response element (cARE) is a 6 bp inverted repeat, 5′-AGAACA-3′, with 3 bps intervening (15). cAREs drive most direct AR target genes and are also recognized by glucocorticoid, progesterone and mineralocorticoid receptors (GR, PR, MR). Receptor- and tissue-specific actions are largely determined by interaction with nonreceptor factors (16,17). Selectivity is also evident at the DNA level since certain elements are activated exclusively by AR but not by other steroid receptors (17). Genomic data reveals that the selective ARE (sARE) is a half-site of the cARE (18). AR homodimers bind this element because of AR’s strong dimer interface and less stringent sequence requirements for the 3′ hexamer. Inability to activate an sARE leads to incomplete virilization in male mice (19) and produces a more oncogenic AR (20), hinting at a pro-differentiative role of sAREs. Although paradoxical that a half-site confers more specific response, numerous examples show weak transcription factor binding sites permit more precise regulation (21). Weak sites allow greater specificity and response over a broad range of inducer, in part due to cooperative interactions with self (as we characterized for clustered full- and half-site AREs (16)) or with other transcription factors (7). Thus, the ‘relaxed response element stringency’ of AR relies on DNA-binding partners, such as FOXA1, to achieve precise, strong activation (17,18). The differential action of sAREs vs. cAREs led us to hypothesize that these elements underlie functional distinctions, with sARE-driven genes being pro-differentiative and cARE-driven genes being pro-proliferative. This underlies our rationale for seeking selective modulators of AR that repress cARE- but not sARE-driven gene expression for PCa treatment.

We developed a high-throughput screen for compounds that elicit differential AR regulation from distinct cARE or sARE elements, similar to a screen for differential modulators of GR (22). Surprisingly, the strongest hit was doxorubicin (dox), which is known to intercalate into DNA and is one of the earliest chemotherapeutics. Dox inhibited AR action on cARE-reporter genes to a greater extent than that on sARE-reporters, and led to greater disruption *in vitro* of AR binding to cARE than sARE DNA. In PCa cells, dox at low dose increased expression of endogenous sARE-driven genes but repressed cARE-driven genes, and AR was differentially recruited to the chromatin of these genes. Since few genes with sAREs have been characterized, we considered genes sARE-like if their expression increased rather than decreased with low dose dox. The differential effect of low dose dox on gene expression was detectable in xenograft tumors. Dox dosage determined global differentiative versus proliferative patterns of gene expression by RNA-seq analysis and distinct landscapes of AR binding in ChIP-seq. The dose-dependent response to dox underscores that AR distinguishes selective from consensus response elements, the importance of these elements in driving functionally distinct patterns of gene expression, and an ability to differentially modulate AR activity on these elements. These results provide a basis to develop new PCa therapies that modulate rather than block AR activity, thus delaying resistance and producing fewer side effects.

## MATERIALS AND METHODS

### High-throughput compound screen

Screens were performed in the University of Michigan Center for Chemical Genomics (CCG). HeLa-A6 cells (a gift from E.M. Wilson (23)) served as useful hosts since they have high AR levels from an integrated expression vector, an integrated PSA-luciferase gene to serve as a positive control for androgen induction, and the ability to withstand manipulations necessary for screening such as washing and replating. In cARE and sARE reporter plasmids, the consensus and non-consensus hormone response elements (HRE3 and HRE2, respectively) of the androgen-specific sex-limited protein (Slp) gene enhancer drove luciferase in the pGL3-basic plasmid backbone (16,20,24). The cARE reporter contained three copies of HRE3 and the sARE reporter four copies of HRE2, to compensate for the more androgen-specific but weaker sARE relative to the cARE. To create the fluorescent protein reporters, the luciferase genes in the pGL3-basic backbone were replaced by fluorescent protein (FP) genes that were PCR-amplified from FP expression vectors using primers allowing insertion into unique restriction sites of the ARE plasmids. Screens were performed in saturating levels of the synthetic androgen R1881 so that a compound interacting with any AR domain would be scored, allowing detection of competitive and noncompetitive inhibitors. Transfection was optimized for maximal separation of activation by R1881 vs. inhibition by bicalutamide. Detailed screen methods are available from DMR.

### Cell culture, viability and transfection assays

CV-1, HEK-293T, LNCaP, C42B and LAPC-4 cell lines were from American Type Culture Collection (Manassas, VA, USA) and were validated, grown and transfected as previously described (20,25). For viability assays, cells were seeded in 96-well plates at 2000–10 000 cells/well in a total volume of 100 μl media containing 10% FBS. Compounds in 100 μl media were added to the cells 12 h later. Following 7 days of incubation, cell viability was assessed by Cell-Titer GLO (Promega, Madison, WI, USA). The values were normalized, and IC:50 was calculated using GraphPad Prism 6 software. R1881 and testosterone were purchased from Sigma-Aldrich (St. Louis, MO, USA) and stored at −20°C in ethanol. Doxorubicin was from AK Scientific (Union City, CA, USA).

### *In vivo* tumor growth

CB17 scid mice from an in-house colony at the University of Michigan were surgically castrated and concurrently implanted with silastic tubing containing 25 mg testosterone for sustained release. After 1–1.5 weeks of allowing circulating testosterone levels to equilibrate to approximate human hormone levels, 3 × 10^6^ LAPC4 cells in matrigel were injected into both flanks of the mice (26). Tumors were detected by palpation and growth followed by caliper measurement twice weekly. Mice were assigned to dox dose groups (0, 0.5, 1.7 and 5.0 mg/kg) when tumors reached 200 mm^3^ in size (calculated as [length × width^2^]/2), or in the case of mice with no palpable tumors, 3 weeks after grafting. Dox was prepared in 0.9% sterile saline and administered twice weekly with 100 μl intraperitoneal injections. The highest dose, similar to what is used in man, proved toxic. Mice were euthanized, and tumors harvested when the tumors reached about 2 cm^3^ in size, or 3 weeks after treatment initiation. At euthanasia, tumors were resected and portions stored in RNA*later* Solution (Invitrogen), 10% formalin or snap frozen in liquid nitrogen for future analysis.

### RNA isolation, reverse transcription and qPCR

Total RNA was isolated from cells or homogenized xenograft tissue stored in RNA*later*. Solution was removed from −80°C and thawed, and RNA was isolated using the miRNAeasy kit, including the optional DNAse digestion (Qiagen, Valencia, CA). cDNA was synthesized from 1000 ng total RNA using Maxima First Strand cDNA Synthesis III Kit for RT-qPCR (Thermo Fisher Scientific), and/or the High-Capacity cDNA Reverse Transcription Kit (Applied Biosystems). Isolated RNA was quantified and checked for integrity and purity (260:280 ratio) using NANOdrop Spectrophotometer (Thermo Scientific). Quantitative real-time PCR was performed in triplicate using standard SYBR green reagents and protocols on a StepOnePlus Real-Time PCR system (Applied Biosystems). The target mRNA expression was quantified using the ΔΔCt method and normalized to β-actin expression. All primers (Supplemental Table S1) were designed using Primer 3 (http://frodo.wi.mit.edu/primer3/) and synthesized by Integrated DNA Technologies (Coralville, IA).

### RNA-sequencing

LNCaP cells were cultured in charcoal-stripped serum (CSS) and then treated 24 hrs with vehicle, 1 nM R1881, or R1881 plus 0.1, 0.4 or 0.7 μM dox. RNA-seq was performed on RNA extracted as above, using the Illumina HiSeq 2000 in paired end mode, as previously described (27). For each gene, a rank list was generated by ordering each gene in the differential expression analysis by the DESeq2 log_2_ fold change (FC) value (28). These rank lists were used in a weighted, pre-ranked GSEA analysis against MSigDBv5 (29,30). Significant associations were determined for any gene set having an FWER *P*-value <0.01. More complex statistical designs probing for the interaction between dox treatment (at different doses) under R1881-treated conditions, were formulated as linear models and fit using Limma (31).

### ChIP-qPCR and ChIP-seq

The ChIP assays were performed using a HighCell ChIP kit with IPure 2.0 kit elution (Diagenode) according to the manufacturer's protocol. The antibodies used for ChIP assays are AR_PG-21 (Millipore Cat. # 06-680); TBP (Abcam Cat. #ab63766), and Normal Rabbit IgG Control (Diagenode). LNCaP cells were grown in charcoal-stripped serum containing media for 48 h followed by 12 h of vehicle or 1 nM R1881 and dox treatment (0.1 μM for ‘low,’ 0.4 μM for ‘medium,’ and 0.7 μM for ‘high’) for all ChIP-qPCR and ChIP-seq conditions. ChIP-seq sample preparation for sequencing was performed according to the manufacturer's instructions (Illumina). Further experimental details as well as information on ChIP-seq enrichment analysis and overlaps of AR binding are in Asangani *et al.* (32). Transcriptional motifs were identified using MEME or DREME (33).

### Statistical analyses

Data was analyzed using GraphPad Prism software version 7.0 (GraphPad Software, La Jolla, CA, USA). EMSA quantification and qPCR experiments were performed in triplicate to determine mean standard error, and are representative of repeated experiments of multiple biological replicates. Student's t-tests were performed with normalization to control analyses to obtain *P*-values between individual conditions, and used a one-way ANOVA with Pair-wise Multiple Comparison Procedures (Holm–Sidak method) across multiple conditions. Growth data was analyzed using multiple pair-wise Student's *t*-tests.

## RESULTS

### High-throughput response-element-selective compound screen for modulators of AR transcriptional activity

**Figure 1.**
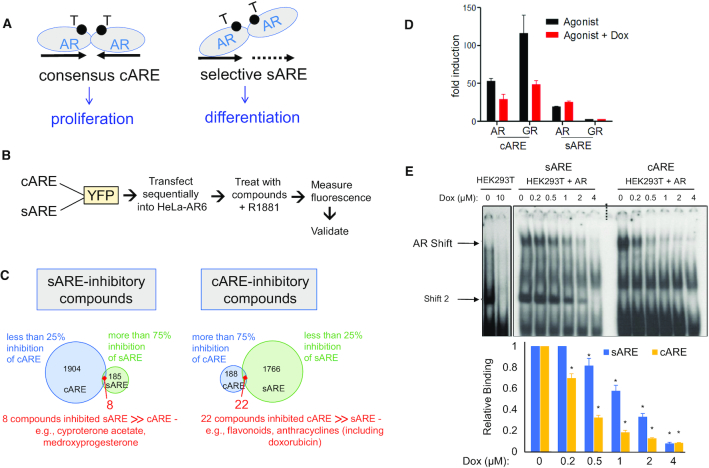
Screen for compounds that act as differential modulators of AR activity, nomination of doxorubicin, and validation of AR response element selectivity. (**A**) Schematic illustrating the canonical AR palindromic repeat response element and selective AR response element comprised generally of half sites, both elements bound by head-to-head AR dimers. These elements are hypothesized to favor pro-proliferative versus pro-differentiative gene expression. (**B**) Flow chart outlining experimental steps of compound screening in HeLA-A6 cells. (**C**) Venn-diagram of compounds identified in the screen that specifically inhibit sARE (>75% inhibition of induced signal, with <25% inhibition of cARE signal) or cARE (>75% of signal, with <25% inhibition of sARE signal). (**D**) cARE or sARE driven reporters were transiently co-transfected with AR or GR expression plasmids into CV-1 cells. Cells were starved 24 h in 2.5% charcoal-stripped serum (CSS) and then treated with the respective AR or GR agonists (1 nM R1881 or 1 μM dexamethasone) with 0.4 μm dox or DMSO for 24 h prior to dual luciferase assay. Shown is the average of duplicates of a representative transfection. (**E**) Protein–DNA interaction was determined by electrophoretic mobility shift assays (EMSA), performed with 5 μg nuclear extracts from AR-transfected HEK293T cells and 1 ng ^32^P-cARE or -sARE oligo probes. Reactions were mixed on ice for 10 min with varied dox concentrations and then complexes separated by electrophoresis. Top: the specific AR-ARE shift, confirmed by antibody supershift (not shown), is denoted by an arrowhead; shift 2 appears in the absence as well as presence of AR and thus is due to non-AR factors. Bottom: histogram indicating image density from scans of three independent experiments. Error bars represent standard deviation (SD) (* indicates *P* < 0.05 compared to both the relative control and within each condition).

We developed a screen to test whether genes related by function may be co-regulated by distinct androgen response elements (AREs), which could account in part for differences in genes regulated by AR in normal or early disease prostate cells from those in castration-recurrent PCa (CRPC) (13). While most AR target genes utilize inverted repeat consensus AREs (cAREs), more selective, AR-specific regulation relies on half-sites (sAREs) or nonconsensus elements (14,18). We hypothesized that pro-proliferative genes required cAREs while pro-differentiative functions involved sAREs (Figure 1A). To treat cancer and its side-effects, we sought compounds that would repress activation of cAREs but not sAREs.

Differential AR activation was assessed by transient transfection of fluorescent reporter genes driven by multimerized cAREs or sAREs into HeLa-A6 cells that have high AR levels from a stably integrated expression vector (23) (Figure 1B). Citrine (YFP) had the best signal-to-noise ratio and was thus used in sequential screening, first for inhibitors of cARE-YFP activation and then for inhibitors of sARE-YFP. We initially screened the Spectrum FDA-approved library, a collection of over 2000 small molecules, and the NIH library of 450 compounds used in clinical trials. Of these compounds, 8% suppressed the activation of AR-driven promoters by more than 75%, and most of those suppressed cAREs and sAREs equivalently. Known anti-androgens (cyproterone acetate, medroxyprogesterone) were identified, indicating screen validity (Supplemental Table S2). To assess selectivity, compounds that suppressed one ARE by >75% but the other by less than 25% were tallied (Figure 1C). Twenty-two compounds strongly suppressed cARE activity but had minimal effect on sAREs, and curiously, several of these were anthracyclin antibiotics (Supplemental Table S3, anthracyclins highlighted in red), including the commonly utilized chemotherapeutic doxorubicin (dox). Five of these compounds had acceptable inhibition curves and potencies in dose response assays (data not shown) to proceed with validation.

Fresh powder samples of the five leads were tested for inhibition of cARE- or sARE-driven luciferase reporters transiently transfected into CV-1 cells. One compound, dox, suppressed cARE but not sARE activation by AR plus R1881 ligand (Figure 1D). Dox also inhibited GR activation via the cARE (GR does not activate the AR-selective sARE), the first indication that the differential effect of dox depended on the response element rather than the receptor. Since the screen contained high levels of reporter DNA as well as AR protein, the response element itself was a plentiful target. Dox is known to intercalate into DNA with some sequence selectivity, which disrupts topoisomerase II action, causing DNA double strand breaks and activation of the DNA damage response (DDR) (34). Here, dox provided proof-of-concept that the transcription-based screen detected differences between AREs modeling distinct promoter signatures.

Given the remarkable finding of dox as a selective AR modulator, we screened an additional 7600 compounds from ChemDiv 100K, Prestwick, LOPAC, MS2400 and Biofocus natural products libraries. There were 124 compounds that showed >70% inhibition of cARE-citrine activity and <50% cell toxicity. Of these, 109 compounds had reasonable dose response, and 15 inhibited cARE- but not sARE-citrine expression. One of these compounds was also a topoisomerase inhibitor that intercalates into DNA, strengthening the notion that a DNA response element sequence difference could differentially modulate AR signaling. However, none of these additional hits were readily amenable to further development.

To confirm that dox exerted a differential effect on AR due to the response element, we examined protein–DNA interaction by electrophoretic mobility shift assay (EMSA) (Figure 1E). HEK293T nuclear extracts, with or without overexpressed AR, were incubated with cARE or sARE oligonucleotides and complexes were separated by electrophoresis. Antibody super-shift confirmed specificity of the AR complex, whereas ‘Shift 2’ was nonspecific, occurring regardless of the presence of AR. The AR shift appeared weaker with the sARE than cARE probe in the absence of dox, as expected due to the weaker affinity of AR for sAREs than for cAREs (35). As the dox concentration was increased, binding of AR to the cARE decreased more than to the sARE (Figure 1E), indicating greater sensitivity of the AR-cARE complex to destabilization by dox intrusion. Although there are two contact sites for AR monomers in the cARE compared to the sARE, dox may induce different conformations depending on DNA sequence, with AR binding more stringently to the cARE and more flexibly to the sARE.

### Dox concentration differentially influences AR-dependent gene regulation

**Figure 2.**
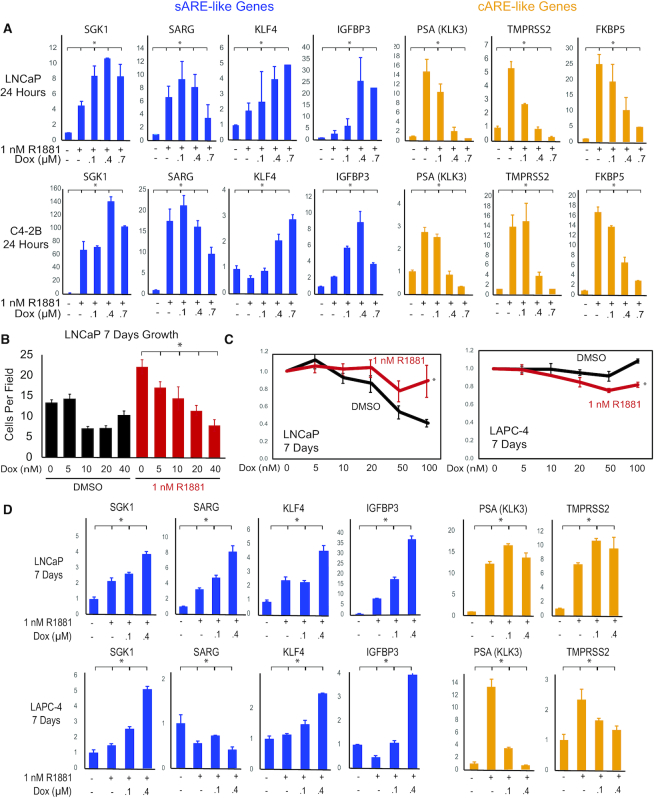
*In vitro* validation of dox-mediated induction of sARE-like AR target genes and inhibition of cARE-like AR target genes. (**A**) LNCaP and C42B cells were plated in six-well plates in RPMI 1640 medium with 10% FBS for 2 days, then hormone-starved in 2.5% CSS for 24 hrs prior to treatment with 1 nM R1881 alone or with varying dox concentrations for 24 h. Q-RT-PCR analysis of total RNA extracted from cells was used to quantify gene expression. Genes indicative of sARE response are *SGK1*, SARG (*C1orf116*), *IGFBP3*, and *KLF4*; genes representing the cARE pattern of response are PSA (*KLK3*), *TMPRSS2*, and *FKBP5*. (**B**) Dose response of *in vitro* growth (cell count, *n* = 10, error bars indicate SD) of LNCaP cells treated with increasing concentrations of dox, with (red) or without (black) hormone treatment (1 nM R1881) for 7 days. (**C**) Cell growth was assayed for 5 × 10^3^ LNCaP or LAPC4 cells seeded per well in 96-well plates in complete media, then starved 2 days in CSS prior to treatment as indicated (black, no R1881; red, 1 nM R1881; dox concentration is shown at bottom). Cells were harvested and MTT assays (*n* – 5) performed after 7 days. Proliferation relative to control is plotted; error bars indicate SD. (**D**) Q-RT-PCR analysis of total RNA extracted from LNCaP and LAPC-4 cells under the same growth conditions as in A to quantify gene expression after a 7-day treatment with R1881 (1 nM) alone or with varying dox concentrations. Fold-changes were normalized to β-actin and data plotted relative to the average of untreated control with error bars representing SD (* indicates *P* < 0.001).

We next examined endogenous AR target gene expression in multiple prostate cell lines to determine to what extent dox affected AR activation of sAREs versus cAREs on natural promoters, and whether dox treatment distinguished genes involved in differentiation or survival from those involved in oncogenesis. Analysis was restricted to cells with endogenous AR, including LNCaP, LAPC-4 and C4-2B cells, which model early, mid, and late-stage PCa, respectively. The first two lines depend on androgen for growth, whereas C4-2B is androgen-independent in that AR acts regardless of hormone to direct a gene expression program similar to that of CRPC (36). Genes tested were regulated by AR or were candidates for AR interaction in DNA damage response (DDR). LNCaP and C4-2B cells were treated for 24 hrs with 1 nM R1881 in the absence or presence of increasing concentrations of dox (Figure 2A). Most AR targets, including classic prostate markers PSA (*KLK3*), TMPRSS2, and FKBP5, were inhibited by dox in a dose-dependent manner and have well characterized cAREs. In contrast, genes that increased expression above hormone-induced levels with low dose dox were called ‘sARE-like’. SGK1 and SARG (Specifically Androgen-Regulated Gene, *C1orf116*) are prototypical sARE-driven genes, identified by their altered expression in mice bearing a mutated AR that cannot activate sAREs (18,19). SGK1 is affected by both AR and GR and enhances cell survival (37), whereas a function for SARG is not yet known. KLF4, a tumor suppressor gene acting in part through P21-dependent cell cycle arrest (38), was moderately responsive to R1881 in LNCaP cells, less so in C4-2B cells, and upregulated by low-dose dox; it is thus designated sARE-like although response elements have not been determined. Similarly, IGFBP3 was sARE-like in its upregulation by low-dose dox and potential anti-tumor effects (39,40). Numerous AR targets were tested for upregulation by low dox but relatively few showed this behavior, in accord with few genes identified as dependent on sAREs in the AR mutant mice (19).

To determine to what extent differential effects of dox might affect tumorigenesis, we examined long-term dox effects on cells (Figure 2B, C). While higher concentrations of dox were toxic, both LNCaP and LAPC4 cells withstood low dose treatment for at least one week. In LNCaP cells (Figure 2B), 5 nM dox selectively slowed AR-induced proliferation, and higher concentrations of dox slowed proliferation regardless of R1881 with significant cell death. Concentrations up to 100 nM dox inhibited proliferation of LNCaP cells in the absence of R1881, likely reflecting androgen-dependency rather than dox toxicity, but dox effects in the presence of R1881 were modest. In LAPC4 cells, dox was somewhat more inhibitory in the presence than absence of R1881, and was not significantly toxic.

Following 7 days of dox treatment (Figure 2D), enhancement of AR induction at low dox doses was evident for genes with known sAREs (SGK1, SARG) and genes with similar regulation (KLF4, IGFBP3). The inhibitory effect of dox on the expression of cARE-driven genes was less pronounced, particularly in LNCaP cells, perhaps due to differences in hormone levels, disease stage modeled, or secondary effects. These results show that the selective effect of dox occurs at low drug doses, where cARE-driven gene expression is inhibited while sARE-driven genes are upregulated, and is sustained over time.

### Dose-dependent differential effects of dox in mouse xenograft tumors

**Figure 3.**
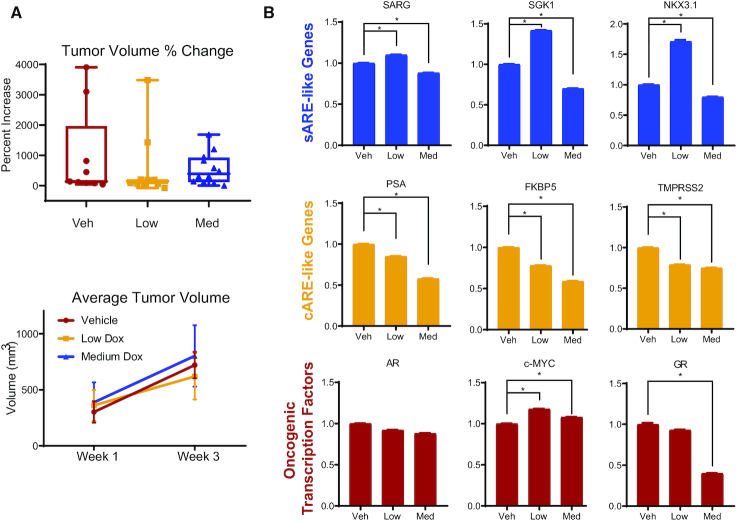
*In vivo* validation of dox-mediated induction of sARE-like AR target genes, and inhibition of cARE-like AR target genes. (**A**) Mouse xenograft tumor growth was assessed following dox treatment. 10^6^ LAPC4 cells were inoculated into flanks of castrated mice bearing testosterone pellets that maintained human physiologically relevant levels of the hormone. Tumors were followed by palpation and measured by calipers, with volume calculated as (length × width^2^)/2. Tumor volume change (top) and average tumor volume (bottom) represent tumor growth in mice treated for 3 weeks with either no (vehicle control – veh, *n* = 7), low (*n* = 12), or medium (med, *n* = 12)) dox doses (0, 0.5, 1.7 mg/kg, respectively). Growth was heterogeneous, but as shown on the lower left, trended to slower growth for the low dox dose, compared to untreated or treated with higher dox doses. (**B**) Total mRNA extracted from xenograft tumors at endpoint was subjected to Q-RT-PCR for the mRNAs of sARE responsive genes *SGK1*, SARG (*C1orf116*) and *NKX3-1*, cARE responsive genes PSA (*KLK3*), *FKBP5* and *TMPRSS2*, as well as oncogenic transcription factors *AR*, *MYC*, and GR (*NR3C1*). Fold-changes were normalized to β-actin and data plotted relative to the average of untreated control with error bars representing SD (* indicates *P* < 0.05).

To determine whether dox selectivity affected PCa progression, mouse xenograft tumors from LAPC4 cells were established in CB17-SCID mice. Mice were treated after tumor detection with 0, 0.5 (low) or 1.7 (medium) mg/kg dox delivered twice weekly, corresponding to about one-tenth and one-third human doses, respectively (Figure 3). A high dose of dox was toxic to mice. Tumors grew heterogeneously, whether in untreated or dox-treated mice (Figure 3A), and faster than anticipated. Pools of tumor RNAs were subjected to qRT-PCR to examine whether low dose dox promoted less proliferative, more differentiated growth (Figure 3B). Remarkably, despite tumor heterogeneity in size and growth, gene expression in these samples was consistent within treatment groups, with sARE-like genes upregulated with low dox and cARE-driven genes inhibited in a dose-dependent manner. NKX3.1, a known AR-regulated transcription factor essential for prostate differentiation and development (41), also illustrated an sARE-like response to dox in vivo. AR mRNA levels did not change with treatment, but c-MYC expression was significantly higher with low dose dox and somewhat higher with medium dox. GR mRNA was substantially lower with medium dox, suggesting that the changes in gene expression are unlikely to be mediated by GR compensating for AR. Thus, at the molecular level, these tumors evidenced the differential dose-dependent effects of dox.

### Low vs. high dose dox treatment produces differentiative versus oncogenic gene expression patterns

**Figure 4.**
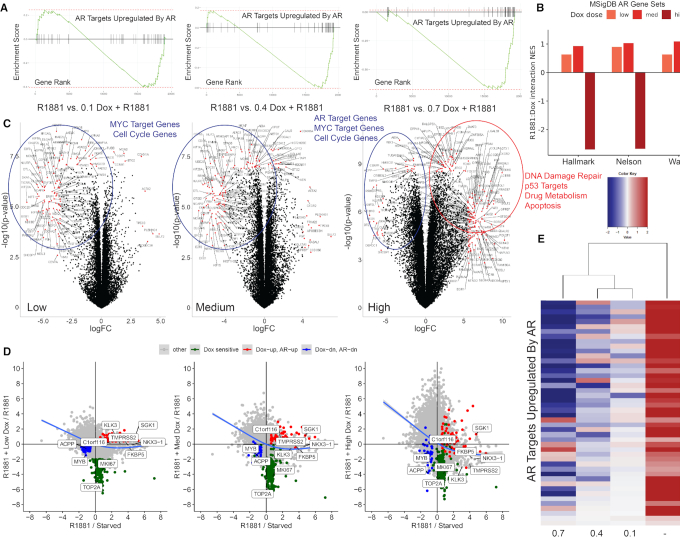
RNA-sequencing data confirms the anti-proliferative effect of low dose dox in LNCaP cells. (**A**) Gene set enrichment analysis (GSEA) plots showing repression of the *Androgen Receptor Targets Upregulated by Androgen Receptor* gene set shown for low (0.1 μM), medium (0.4 μM) and high (0.7 μM) dox conditions. (**B**) Effect of low (0.1 μM), medium (0.4 μM) and high (0.7 μM) dox on the expression of three independent AR target gene sets obtained from the Molecular Signatures Database (MSigDB), under R1881 conditions. (**C**) Differentially expressed genes from RNA-seq data were visualized by volcano plots, for conditions noted above each plot. Genes labeled and marked by red dots were most significant (*P*-value < 10^−7^) with fold changes above the cut-off [abs(Fold Change) > 4]. Top significantly relevant pathways identified by GSEA are highlighted through upregulated (red) or downregulated (blue) genes encircled on plots. (**D**) Scatter plots illustrating the impact of dox at different dosages relative to expression changes following androgen stimulation. Androgen stimulated genes (1 nM R1881 stimulated compared to charcoal stripped serum starved condition) plotted on x-axis plotted against response to androgen stimulation in the presence of low (0.1 μM – left panel), medium (0.4 μM – middle panel), and high (0.7 μM – right panel) dox treatment. Overall trend indicated by blue line. The most downregulated genes by dox treatment indicated with green dots (dox sensitive). Genes induced by both androgens and dox indicated with red dots (Dox-up, AR-up), and the most downregulated genes by both androgens and dox indicated with blue dots (Dox-dn, AR-dn). All other genes shown as gray dots (other). (**E**) Heat map and unbiased clustering of differentially AR-regulated genes identified from the *Androgen Receptor Targets Upregulated by Androgen Receptor* gene set in LNCaP cells treated with 1 nM R1881 and low (0.1 μM), medium (0.4 μM) or high (0.7 μM) dose dox.

To view how global gene expression varied with dox concentration and to discover additional sARE-like genes, transcriptome analysis was performed for LNCaP cells (Figure 4). The major effects of dox treatment on AR regulation were analyzed for the gene set ‘AR targets upregulated by AR’ (32) (Figure 4A) and Molecular Signatures Database (MSigDB) AR pathways (Hallmark, Nelson (42), and Wang (13) gene sets) (Figure 4B), as well as the effect of dox on individual genes (Figure 4C). Compared to CSS, R1881 treatment massively induced numerous genes, many of which are canonical AR targets (e.g., TMPRSS2, KLK2/3, FKBP5; Supplemental Figure S1). Overall, there were slight changes to global AR signaling with lower doses of dox, but consistently large decreases seen with high dox. In the presence of androgen, only high levels of dox affected AR expression (Supplemental Figure S1A). When 0.1 μM dox was added to R1881, there were subtle differences but the major effect, when compared to cells in CSS, was on androgen stimulation. However, when compared to R1881-stimulated (control) cells, the addition of 0.1 μM dox led to pronounced downregulation of many genes, including cell cycle control genes, c-MYC targets, and genes that promote proliferation (e.g. CENP, MCM, BUB, TOP2A) (Figure 4C, control versus 0.1 dox); some genes were upregulated, particularly those involved in signaling by the tumor suppressor p53 (e.g. MDM2, CDKN1A, BTG). Although major differentiative signatures were not obvious, re-expression of cell cycle arrest genes indicated a strong anti-proliferative effect.

The addition of 0.4 μM dox (Figure 4C, middle panel) decreased expression of androgen targets and induced genes involved in DDR, lipoprotein signaling, and cholesterol metabolism, with MYC targets downregulated and cell cycle genes dysregulated, thus confirming the LAPC-4 xenograft tumor results (Figure 3B). At 0.7 μM dox (Figure 4C, right panel), there was further differential expression for genes involved in drug metabolism, cell cycle, apoptosis, as well as p53 and MYC signaling. Most significant was the nearly abolished expression of AR-regulated genes. The gene expression profile with 0.1 μM dox was similar to that with R1881 alone but as dox increased, the profile showed dramatic downregulation of AR targets.

Comparing dose-dependent effects of dox on androgen regulated gene expression helped to distinguish effects due primarily to dox rather than dox modulation of androgen induced genes (Figure 4D, Supplemental Figure S1). Normalized RNA-seq data (Figure 4D) showed that genes induced the most by dox alone with positive y-values were also often the most induced by androgen (red dots), and those the most inhibited by dox with negative y-values were cell cycle genes (green dots). This analysis further illustrated the strong anti-proliferative effects of dox, since the most downregulated genes at all dox concentrations were cell cycle genes, also identified in Figure 4C (TOP2A, MKI67). However, most AR-upregulated genes were less sensitive to the global dox-induced downregulation of gene expression. In fact, several sARE-like genes (SGK1, C1orf116; Supplemental Figure S1B, C) were further induced by dox treatment. We also identified a set of genes repressed by androgens that were further decreased in expression by dox treatment (blue dots – MYB, ACPPP), suggesting that a response-element specific effect of dox may also play a role in AR-mediated transcriptional repression. This dox-specific modulation of AR-mediated transcriptional repression is further evidenced by the fact that many androgen-repressed genes showed relative upregulation with dox treatment (Figure 4D, Supplemental Figure S1D).

A further indication that low dose dox treatment led to differential biology and was not simply sub-toxic was seen by displaying differentially affected genes across treatment groups as a heat map with nonbiased clustering (Figure 4E, Supplemental Figure S2). The low and medium doses were most similar, flanked by mostly downregulated genes of high dose dox and upregulated genes of androgen without dox (See Supplemental Figures S3–S7 for enrichment scores and pathway heat map). Some of the genes that maintained expression in low dox similar to that with androgen included NCAPD3 and CENPN, which are involved in chromosome separation, and ENDOD1, an endonuclease involved in prostate tumor suppression, suggesting interesting candidates of low dox effects that may restore normal function of AR. Overall, the dox dose response demonstrated a pause in cell growth at 0.1 μM, a switch to increased oncogenic growth at 0.4 μM, and the appearance of additional cancer pathways and cell toxicity by 0.7 μM dox.

### Dox affects the AR chromatin landscape in a dose-dependent manner that highlights response element differences

**Figure 5.**
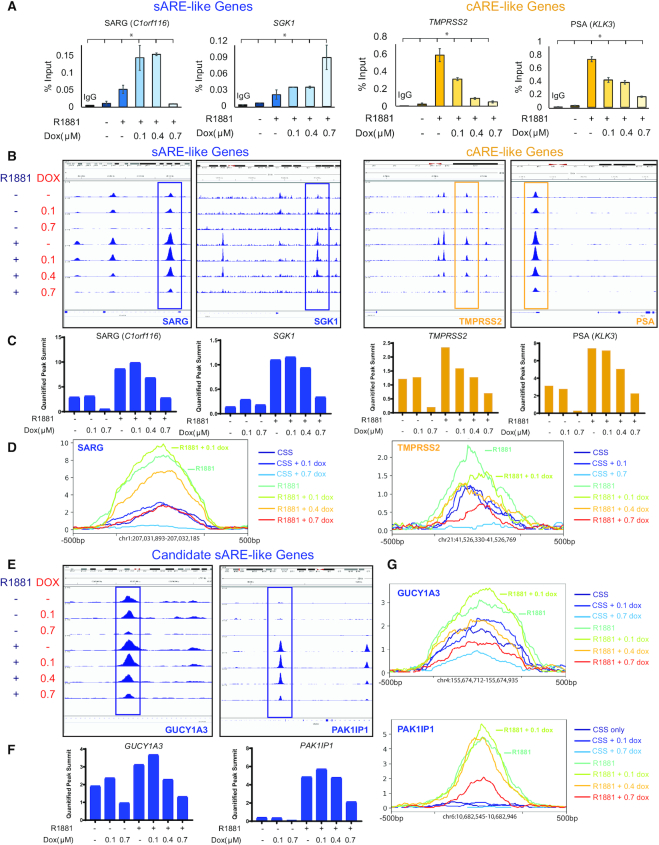
Dox differentially affects AR recruitment to chromatin, enhancing sARE binding and decreasing cARE binding at low concentrations. (**A**) ChIP-PCR in LNCaP cells with AR antibodies, IgG control, and purified DNA was quantified by qPCR. Left (blue) – qRT-PCR targeting promoter regions of cARE-driven PSA (*KLK3*) and *TMPRSS2*. Right (yellow) - qRT-PCR targeting sAREs in the promoter of *SGK1* and intron 1 of SARG (*C1orf116*) (* indicates *P* < 0.001). (**B**) Corresponding ChIP-seq peaks, including those analyzed by qPCR, visualized by Integrative Genomics Viewer (IGV) (60). Peaks that are boxed are enhanced by low-dose 0.1 μM dox in the sARE-like genes of *SGK1* and SARG (*C1orf116*), and show a dose-dependent decrease in the cARE-like genes PSA (*KLK3*) and *TMPRSS2*. (**C**) Read count values for the summits of each peak illustrated by boxes in B graphed for each ChIP condition (quantified peak summits). (**D**) Isolated peak traces from peaks boxed in part B graphed together on the same axis to illustrate relative area under the curve. (**E**) ChIP-seq peaks of candidate sARE-like genes identified by RNA-seq, *GUCY1A3* and *PAK1IP1* visualized by IGV, with sARE-like peaks boxed as in part B. (**F**) Read count values for the summits of each peak illustrated by boxes in E graphed for each ChIP condition (quantified peak summits). (**G**) Isolated peak traces from peaks boxed in part E graphed together on the same axis.

In order to link the effect of low dose dox on gene expression to underlying differences in AREs, we examined AR binding within chromatin. The dox-dependent difference in AR binding to sAREs *vs*. cAREs, as shown by EMSA (Figure 1E) was also observed *in vivo* by chromatin immunoprecipitation (ChIP) analysis (Figure 5). AR binding within enhancer/promoter regions of model sARE (SARG, SGK1) or cARE (PSA, TMPRSS2) genes was first tested by ChIP-qPCR of DNA from LNCaP cells treated with or without R1881 and varying dox concentrations for 24 h (Figure 5A). The ChIP profile mirrored gene expression; specifically, sARE-driven SGK1 and SARG showed increased AR in chromatin with low dox, whereas cARE-driven PSA and TMPRSS2 showed reduced AR in chromatin as the dox dose increased and gene expression decreased. RNA Pol-II binding was similar to that of AR, indicating the effect of dox impacted gene expression in both positive and negative directions (not shown).

To obtain a genome-wide view of differential effects of dox on AR binding, we undertook ChIP-seq analysis using published methods (32). To confirm quality and reproducibility of ChIP-seq results, sequence reads with AR binding peaks were aligned to genome browser views of sARE and cARE genes, with the area of each peak on the ChIP-seq gene tracks correlated with the number of sequence reads (Figure 5B–D). For example, the boxed SARG peaks showed increased height with 0.1 μM dox compared to R1881 alone (compare fourth and fifth tracks down in Figure 5B, and quantified in Figure 5C, D), whereas the TMPRSS2 AR peaks decreased progressively even at low dose dox. Similar analysis was applied to two new candidate sARE genes, GUCY1A3 and PAK1IP1, in Figure 5E–G. Both SARG and TMPRSS2 have multiple AR peaks, and unlike the peak boxed for SARG, or to the right in the PAK1IP1 reads in Figure 5E, the other AR binding sites behaved more like TMPRSS2 cAREs, decreasing with increasing dox. This underscores that genes may be affected by multiple consensus and non-consensus AREs, even within a single binding peak (see Supplemental Figure S8), and use of these elements may vary in a context-dependent manner.

**Figure 6.**
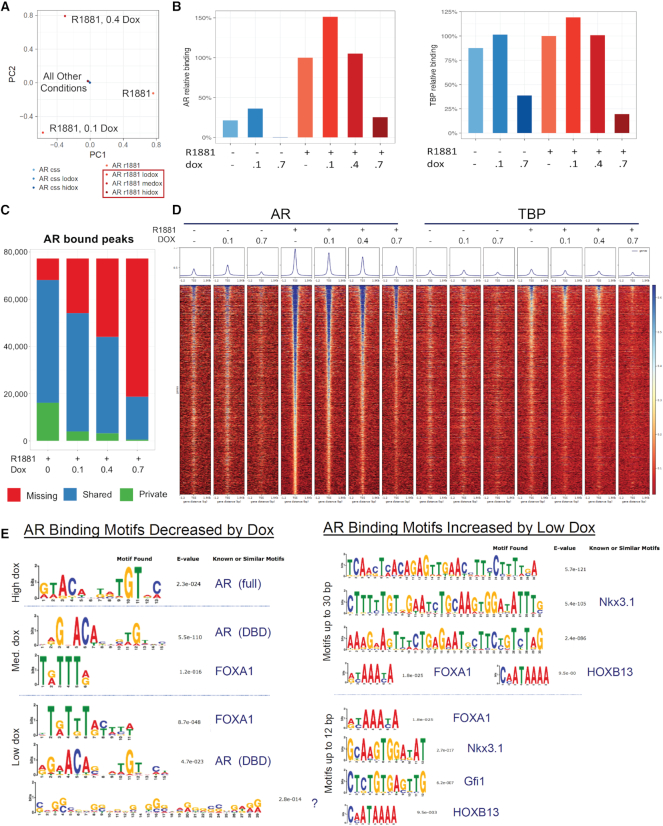
Dox disrupts global AR binding in a dose-dependent manner, and reveals enhanced AR binding motifs in ChIP-seq at low dose dox. ChIP enrichment levels within a peak or site were calculated after sequence alignment to the HG38 reference genome using Bowtie, sorted by NovoSort and duplicates removed with Samtools; additional bioinformatics details are in Asangani *et al.* (32). (**A**) PCA analysis for ChIP-seq conditions with CSS-starved LNCaP cells (AR css) treated with 0.1 μM dox (AR css lodox) or 0.7 μM dox (AR css hidox), or with similar conditions stimulated with 1 nM R1881 (AR r1881) with 0.1 μM dox (AR r1881 lodox), 0.4 μM dox (AR r1881 medox), or 0.7 μM dox (AR r1881 hidox) for 12 hrs. Large differences between all R1881-induced samples, except for 0.7 μM dox (AR r1881 hidox), were seen. (**B**) Relative binding intensity of all peaks from the conditions described in A, with immunoprecipitation of AR (left) or TATA-box binding protein (TBP, right). (**C**) AR bound peaks per treatment for 77 000 unique AR sites sorted to ‘missing’ (red - peaks found in other conditions but not self), ‘shared’ (blue – peaks in common with other conditions), and ‘private’ (green - unique to that treatment – likely background). Data of B and C indicate that low dox increases AR binding at a few sites but reduces binding for most. (**D**) Peak-density heat map of AR and TBP in ChIP-seq conditions. Peak summits were aligned and rank-ordered based on all identified peaks distributed −1.2 to +1.5 kb of the transcription start site. Read-coverage distribution in box above heat map illustrates intensity and coverage of transcription factor binding in each treatment condition. (**E**) Transcriptional motifs were discovered by MEME or DREME for sequence motifs up to 30 bp in length. Motifs shown are for the top 1000 peaks changed by dox. Left: motifs that decreased with high (0.7 μM), medium (med.: 0.4 μM), or low (0.1 μM) dox. Right: motifs that increased with low dox. NOTE: No motifs were found to be significantly increased by medium or high dose dox. *E*-values are indicated, as are factors with known or similar motifs from transcription factor databases.

Dox disruption of AR binding was first globally viewed by principal component analysis (Figure 6A). Samples clustered in the center revealed negligible AR peaks in the absence of hormone and in the high dox sample where transcription is declining and cells are dying. In contrast in the R1881-treated samples, activated AR drove major differences dependent on dox dose. Relative AR binding substantially increased with R1881, and increased further with 0.1 μM dox before declining at higher concentrations (Figure 6B). The control TATA-box Binding Protein (TBP) marked active genes more reliably than Pol II, which also binds inactive enhancers and paused promoters (43). Relative TBP binding showed little effect of dox, except at high levels reflecting toxicity, supporting the notion that the effect of low dose dox was AR-mediated and not a general transcription effect. In Figure 6C, 77 000 unique AR sites in this dataset were divided by their occurrences in different samples, with shared sites (blue segment) indicating dose-dependent loss of AR binding with increasing dox. Because private sites (green segment) did not increase with treatment, our results indicated that low dox did not cause new AR binding sites to appear but rather redistributed AR to increase binding at existing sites. Combined with our ChIP-seq analysis of AR relative binding under various conditions of R1881 and dox (Figure 6B), our results indicated significantly greater AR binding at a small number of genes, without redistribution of AR to new sites within chromatin (Supplemental Figure S9) particularly at lower dox doses.

That the effect of dox is specific for AR and not all transcription factors is evident in the read peak heat maps comparing AR and TBP (Figure 6D). The majority of TBP sites did not depend on androgen and were resistant to the effects of dox, unlike AR binding sites that showed an inverse correlation with dox concentration. Motif analysis showed that the AR-binding sites that were most decreased by high dox relative to R1881 alone were those with a cARE, which was the only motif found with significance (Figure 6E); this finding thus corroborates the effect of high dox in obliterating androgen-induced transcription. At 0.4 μM dox, most decreased sites were AREs but FOXA1 sites were also affected. At low dox, the most abundant motifs among decreased AR sites were for FOXA1, followed by AREs and an intriguing G-rich tract of unknown significance but perhaps reflecting the sequence preference of dox (44). FOXA1 is the well-described pioneer factor for AR binding, with sites in prostate cells frequently near or overlapping AREs (45). A half-site ARE motif was not evident in this approach, reflecting our finding that the sARE was most resistant to dox. Overall, the motifs decreased by dox treatment confirm that binding to AREs is lost with dox in a dose-dependent manner (Figure 6E).

Of greater interest were the AR binding motifs increased by low dose dox (Figure 6E, right). In addition to the sites that were increased, there were those that were maintained, representing peaks most resistant to dox treatment. Remarkably, neither cAREs nor sAREs were apparent, but rather motifs for a number of key prostate-specific AR-interacting factors, such as NKX3.1 and HOXB13, as well as FOXA1. Motifs for Gfi1 were intriguing since this factor represses cell cycle progression, thus adding to the notion that these sites mark genes involved in differentiation rather than proliferation. Overall, the motifs that were increased by low dose dox treatment suggest that AR binding, likely to half-sites, is dependent on key prostate-specific interacting proteins.

## DISCUSSION

Our goal was to redirect AR in prostate cancer to promote differentiation over proliferation by identifying compounds that selectively inhibit AR action on genes driving cancer but not normal growth. Our screen relied on the hypothesis that these genes differ in their response elements, in accordance with genomic studies correlating transcriptional outputs at different PCa stages with differences in AR binding sites (12–14). Most AREs vary somewhat from the canonical sequence shared with other steroid receptors (46), but a few well-characterized elements respond specifically to AR and consist of a half-site working in concert with binding sites for other factors (14,16,18,47). These sAREs may drive functionally distinct gene sets, as demonstrated by discovering that dox elicits differential actions upon intercalation into sAREs versus cAREs that affect tumor progression.

The anthracycline dox, also called Adriamycin, is one of the oldest and still most widely used chemotherapeutics, but the precise mechanism of action remains unclear (48). Dox intercalates into DNA preferentially at 5′-GC and 5′-GG dinucleotides (44), and disrupts base pairing, which in turn disrupts Topoisomerase II action and leads to double strand breaks and activation of the DDR (49). Dox also enhances nucleosome turnover around promoters to affect transcription (50). Recent studies show that AR regulates some genes involved in DDR (51), and cooperates with Topo2a to contribute to prostate cancer progression (52). Therefore, a hormone-DNA repair signaling network makes dox a plausible AR antagonist in prostate cancer (53). Although we had anticipated finding an AR ligand in our screen, the ability of dox to act as an androgen-independent, DNA-dependent AR inhibitor is compelling. Further credence is provided in finding additional anthracyclines and dox analogs as selective hits in both the first (Figure 1C) and second screens. Dox is FDA-approved and sometimes used in late stage PCa. Our findings suggest that it can be repurposed for different applications in treating PCa.

The mechanism of dox action highlights the functional differences in AR response elements, and underscores that AR is prodifferentiative in development and homeostasis but pro-proliferative in oncogenesis. This is modeled by studies in cell lines, which indicate that low dose dox can induce genes with sAREs, like SGK1 and SARG, in contrast to inhibiting classic AR targets such as PSA and TMPRSS2 (Figures 2 and 3). Both EMSA (Figure 1E) and ChIP-qPCR experiments (Figure 5) demonstrate the selectivity of low levels of dox to stabilize AR bound at some sites but not others. Many AR targets have multiple AREs that may differ in sequence, with certain AREs being used at certain times or in certain tissues, thus affecting context-dependent variations in hormone and drug response. Thus, response to dox may depend on the element that is dominant in a given circumstance. For example, only one of the two AREs upstream of SGK1 appears sensitive to low dose dox in ChIP assays (Figure 5B). These different AREs may allow SGK1 to be AR-specific in some contexts but responsive to GR in others (18,37). The global view from RNA-seq (Figure 4) demonstrates the selective effect of low dose dox on a distinct set of target genes. These genes restore more ‘normal’ androgen-regulated growth, allowing departure from the cell cycle and downregulating oncogenic c-MYC signaling, which are all perhaps a prerequisite to further differentiation rather than proliferation (54).

**Figure 7.**
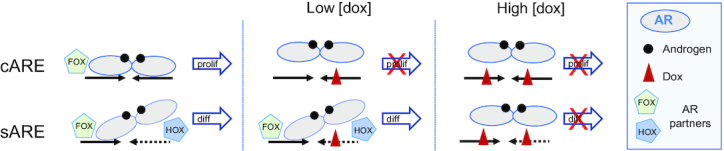
Model of the dose-dependent effects of dox on AR-signaling. AR activity without dox drives both cARE and sARE elements, promoting both proliferation (prolif) and differentiation (diff). With low dox, cARE elements are more sensitive to disruption by dox intercalation into the DNA, while sARE-driven AR activity is maintained or even enhanced by stabilizing interactions with proteins such as FOXA1, HOXB13 and NKX3.1 (FOX, HOX – AR partners), perhaps freed from binding at cARE elements. Low dose dox thus will promote differentiation from sAREs, while inhibiting proliferation at cAREs. Finally, at high doses dox intercalates into more regions of the genome, ultimately disrupting all transcription, including that which is mediated by AR.

The differential effects of dox are emphasized by ChIP-seq results highlighting the role of other factors in distinguishing sARE-like from cARE-like responses (Figure 6). Under low dox conditions, more AR becomes bound to a small set of pre-existing sites. This redistribution likely relies on cooperativity with AR binding partners, particularly FOXA1, HOXB13 and NKX3.1, whose motifs are found by their association with AR, suggesting AR is bound via protein-protein as well as DNA interaction. This motif association underscores a key tenet in gene regulation that ‘weak’ response elements underlie selectivity and specificity of gene control via cooperative interactions with other factors (21,55). Under low dox conditions, reliance on binding partners appears amplified for sARE-like sites, thus leading to higher expression of pro-differentiative genes, but not for cARE genes that are progressively silenced (Figure 7). AR may bind more stringently to the cARE due to structural constraints and be more readily dislodged by dox, whereas contact of one monomer within the AR dimer to the sARE may be more flexible and allow greater influence of binding partners. These effects of dox on AR binding are mirrored in the changes in gene expression, with genome-wide RNA expression confirming that the different global DNA binding patterns produce differentiative vs. proliferative gene signatures, apparent when low dose dox downregulates proliferation and cell cycle genes and maintains expression of known prostate-specific AR gene targets (Figure 4).

An unanticipated clinical opportunity derives from this study. Low dose dox is unlikely to impact late stage PCa, yet the ability to enhance the differential effect between cARE- and sARE-driven genes may favor normal over tumorigenic functions of AR, and may thus be beneficial in watchful waiting or early stage disease. The low dose dox effect cannot reverse aggressive prostate tumor growth, as seen in mouse xenografts (Figure 3), although molecular hallmarks evidence some effect. Further, low dose dox appears to have little efficacy against AR variants noted in castrate-resistant PCa, since there is no effect of dox on growth of 22Rv1 cells that have high AR variant levels until high dox doses are reached (56). Yet low dose dox may exert a modest antiproliferative pressure on normal or pre-neoplastic cells, without significantly reducing cell survival, compounding a cARE/sARE differential achieved in the presence of androgen and AR. The normal drive of AR is towards differentiation, underlying the rationale of intermittent (cyclical) androgen deprivation therapy that is sometimes used after localized PCa treatment to improve patient survival by lessening the side effects of continuous androgen deprivation (57). Further development of this treatment paradigm has led to bipolar androgen therapy in metastatic patients where high concentrations of androgen suppress tumor growth in some models (58). Similar to our findings with low dose dox, high androgen treatment induces DDR, suppresses cMyc activity and induces cell cycle arrest (59). Low dox may be preferable to cyclical androgens in evading a tendency to progress to resistance with long-term treatment. Cardiotoxicity, which is problematic at clinical dox dosages, should be reduced at a low dose and may be avoided altogether with use of dox analogs that are less cardiotoxic. This approach might slow the development of cancer and reduce the pressure on AR to develop treatment resistance.

## DATA AVAILABILITY

GEO accession to all of our data is as follows: GSE137056, https://www.ncbi.nlm.nih.gov/geo/query/acc.cgi?acc=GSE137056.

## Supplementary Material

gkaa178_Supplemental_File

## Notes

Present address: Pia Bagamasbad, National Institute of Molecular Biology and Biotechnology, University of the Philippines Diliman, Quezon City, Philippines.

Present address: Shihan He, ASRC Federal, Morrisville, NC 27560, USA.

Present address: Elizabeth LaPensee, Michigan Institute for Clinical and Health Research, 2800 Plymouth Rd, Ann Arbor, MI 48109, USA.

Present address: Yemi Raji, Department of Medicine, University of Ibadan College of Medicine, Ibadan, Nigeria.
