# Supplementary material for: Differential modulation of the androgen receptor for prostate cancer therapy depends on the DNA response element
Source: Nucleic Acids Res. 2020 Mar 21;48(9):4741–55. doi: 10.1093/nar/gkaa178 (PMC7229860; doi:10.1093/nar/gkaa178)
Supplement: gkaa178_Supplemental_File [file gkaa178_supplemental_file.pdf]

Supplementary Materials for

Differential Modulation of the Androgen Receptor for Prostate Cancer Therapy Depends on the DNA Response Element

Steven Kregel[1,2] †, Pia Bagamasbad[3]†, Shihan He[3], Elizabeth LaPensee[3], Yemi Raji[3], Michele Brogley[3], Arul Chinnaiyan[1,2,4,5,6], Marcin Cieslik[1,2], Diane M. Robins[3,*]

[1]Michigan Center for Translational Pathology, [2]Department of Pathology, [3]Department of Human Genetics, [4]Department of Medicine and Urology, [5]Howard Hughes Medical Institute, [6]Rogel Cancer Center, University of Michigan, Ann Arbor, Michigan 48109, USA

*To whom correspondence should be addressed. Email: drobins@umich.edu

† Joint authors

Supplementary Tables:

Supplemental Table 1: List of Primer Sequences

Primers	Sequence
AR (Exon 1-2)_F	ATCCCAGTCCCACTTGTGTC
AR (Exon 1-2)_R	GGTCTTCTGGGTGGAAAGT
β-actin_F	CACCATTGGCAATGAGCGGTTC
β-actin_R	AGGTCTTTGCGGATGTCCACGT
FKBP5_F	TCTCATGTCTCCCCAGTTCC
FKBP5_R	TTCTGGCTTTCACGTCTGTG
IGFBP3_F	AAAAGCAGTGTCGCCCTTC
IGFBP3_R	TAGCAGTGCACGTCCTCCTT
KLF4_F	GTCTCTTCGTGCACCCACTT
KLF4_R	TGCTCAGCACTTCCTCAAGA
MYC_F	GCTCGTCTCAGAGAAGCTGG
MYC_R	GCTCAGATCCTGCAGGTACAA
NKX3-1_F	CAGTCCCTACTGAGTACTCTTTCTCTC
NKX3-1_R	CACAGTGAAATGTGTAATCCTTGC
NR3C1 (GR)_F	TCTGAACTTCCCTGGTCGAA
NR3C1 (GR)_R	GTGGTCCTGTTGTTGCTGTT
PSA (KLK3)_F	TCATCCTGTCTCGGATTGTG
PSA (KLK3)_R	ATATCGTAGAGCGGGTGTGG
PSA ChIP Primer_F	GCCTGGATCTGAGAGAGATATCATC
PSA ChIP Primer_R	ACACCTTTTTTTTTCTGGATTGTTG
TMPRSS2_F	TGTGGTCCCTTCCAATGCTGTG
TMPRSS2_R	TGCTCATGGTTATGGCACTTGGC
TMPRSS2 ChIP Primer_F	TGGAGCTAGTGCTGCATGTC
TMPRSS2 ChIP Primer_R	CTGCCTTGCTGTGTGAAAAA
SARG (*C1orf116*)_F	AGTCTGAGCCAGCCACAACT
SARG (*C1orf116*)_R	TGTGGATATTCCTAGGGAGG
SARG ChIP Primer_F	CAAGTCTACAGTCTCCCATC
SARG ChIP Primer_R	CTCAAATCCCAGTTTAGCCA
SGK1_F	AGGCCCATCCTTCTCTGTTT
SGK1_R	TTCACTGCTCCCCTCAGTCT
SGK1 ChIP Primer_F	AGGGTTTTCAGGTGGGACAG
SGK1 ChIP Primer_R	TGAGGAGGTAACAAGCGAAGG

Supplemental Table 2: Compounds that suppress sARE (>75%) over cARE (<25%)

Compound	IUPAC name
**Quassin**	2,12-dimethoxypicrasa-2,12-diene-1,11,16-trione
**Oxothiazolidine**	(4S)-2-Oxo-1,3-thiazolidine-4-carboxylic acid
**Cadin-4-en-10-ol**	1,6-dimethyl-4-propan-2-yl-3,4,4a,7,8,8a-hexahydro-2H-naphthalen-1-ol
**Medroxyprogesterone acetate**	[(6S,8R,9S,10R,13S,14S,17R)-17-acetyl-6,10,13-trimethyl-3-oxo-2,6,7,8,9,11,12,14,15,16-decahydro-1H-cyclopenta[a]phenanthren-17-yl] acetate
**Cyproterone acetate**	[(1*S*,2*S*,3*S*,5*R*,11*R*,12*S*,15*R*,16*S*)-15-acetyl-9-chloro-2,16-dimethyl-6-oxo-15-pentacyclo[9.7.0.02,8.03,5.012,16]octadeca-7,9-dienyl] acetate
**Medroxyprogesterone**	(6*S*,8*R*,9*S*,10*R*,13*S*,14*S*,17*R*)-17-acetyl-17-hydroxy-6,10,13-trimethyl-2,6,7,8,9,11,12,14,15,16-decahydro-1*H*-cyclopenta[*a*]phenanthren-3-one
**Coralyne chloride**	2,3,10,11-tetramethoxy-8-methylisoquinolino[2,1-b]isoquinolin-7-ium;chloride
**Irinotecan Hydrochloride Trihydrate**	[(19*S*)-10,19-diethyl-19-hydroxy-14,18-dioxo-17-oxa-3,13-diazapentacyclo[11.8.0.02,11.04,9.015,20]henicosa-1(21),2,4(9),5,7,10,15(20)-heptaen-7-yl] 4-piperidin-1-ylpiperidine-1-carboxylate;trihydrate;hydrochloride

Supplemental Table 3: Compounds that suppress cARE (>75%) over sARE (<25%)

Compound	IUPAC name
**Lorazepam**	7-Chloro-5-(2-chlorophenyl)-3-hydroxy-1,3-dihydro-1,4-benzodiazepin-2-one
**Lofexidine hydrochloride**	2-[1-(2,6-dichlorophenoxy)ethyl]-4,5-dihydro-1*H*-imidazole hydrochloride
**Vecuronium Bromide**	[(2S,3S,5S,8R,9S,10S,13S,14S,16S,17S)-17-Acetyloxy-10,13-dimethyl-16-(1-methyl-3,4,5,6-tetrahydro-2H-pyridin-1-yl)-2-(1-piperidyl)-2,3,4,5,6,7,8,9,11,12,14,15,16,17-tetradecahydro-1H-cyclopenta[a]phenanthren-3-yl] acetate bromide
**Milldurone**	6,7-dimethoxy-3-(6-methoxy-1,3-benzodioxol-5-yl)chromen-4-one
**Cefixime trihydrate**	(6R,7R)-7-[(2Z)-2-(2-amino-1,3-thiazol-4-yl)-2-[(carboxymethoxy)imino]acetamido]-3-ethenyl-8-oxo-5-thia-1-azabicyclo[4.2.0]oct-2-ene-2-carboxylic acid trihydrate
**Isosafrole**	5-(Prop-1-enyl)benzo[d][1,3]dioxole
**N-Methylanthranilic acid**	2-(methylamino)benzoic acid
**Epirubicin hydrochloride**	(7*S*,9*S*)-7-[(2*R*,4*S*,5*R*,6*S*)-4-amino-5-hydroxy-6-methyloxan-2-yl]oxy-6,9,11-trihydroxy-9-(2-hydroxyacetyl)-4-methoxy-8,10-dihydro-7*H*-tetracene-5,12-dione;hydrochloride
**Pergolide mesylate**	(6*aR*,9*R*,10*aR*)-9-(methylsulfanylmethyl)-7-propyl-6,6*a*,8,9,10,10*a*-hexahydro-4*H*-indolo[4,3-fg]quinoline;methanesulfonic acid
**Spaglumic acid**	(2*S*)-2-[[(2*S*)-2-acetamido-3-carboxypropanoyl]amino]pentanedioic acid
**Doxorubicin Hydrochloride**	(7*S*,9*S*)-7-[(2*R*,4*S*,5*S*,6*S*)-4-amino-5-hydroxy-6-methyloxan-2-yl]oxy-6,9,11-trihydroxy-9-(2-hydroxyacetyl)-4-methoxy-8,10-dihydro-7*H*-tetracene-5,12-dione;hydrochloride
**beta-Caryophyllene**	(1R,4E,9S)-4,11,11-trimethyl-8-methylidenebicyclo[7.2.0]undec-4-ene
**Deoxysappanone B 7,3'-dimethyl ether acetate**	[2-methoxy-5-[(7-methoxy-4-oxo-2,3-dihydrochromen-3-yl)methyl]phenyl] acetate
**Rhetsinine**	2-[2-(methylamino)benzoyl]-4,9-dihydro-3H-pyrido[3,4-b]indol-1-one
**Tilorone**	2,7-bis[2-(diethylamino)ethoxy]fluoren-9-one
**Idarubicin hydrochloride**	(7*S*,9*S*)-9-acetyl-7-[(2*R*,4*S*,5*S*,6*S*)-4-amino-5-hydroxy-6-methyloxan-2-yl]oxy-6,9,11-trihydroxy-8,10-dihydro-7*H*-tetracene-5,12-dione;hydrochloride
**Quinamide isopropylidene**	(3*aR*,5*R*,7*R*,7*aS*)-5,7-dihydroxy-2,2-dimethyl-4,6,7,7*a*-tetrahydro-3*aH*-1,3-benzodioxole-5-carboxamide
**Tubaic acid**	4-hydroxy-2-prop-1-en-2-yl-2,3-dihydro-1-benzofuran-5-carboxylic acid
**Bifemelane**	*N*-methyl-4-[2-(phenylmethyl)phenoxy]butan-1-amine
**Veratridine**	[(1*R*,2*S*,6*S*,9*S*,10*R*,11*S*,12*S*,14*R*,15*S*,18*S*,19*S*,22*S*,23*S*,25*R*)-1,10,11,12,14,23-hexahydroxy-6,10,19-trimethyl-24-oxa-4-azaheptacyclo[12.12.0.02,11.04,9.015,25.018,23.019,25]hexacosan-22-yl] 3,4-dimethoxybenzoate
**Azadirachtin**	Dimethyl (2a*R*,3*S*,4*S*,*R*,*S*,7a*S*,8*S*,10*R*,10a*S*,10b*R*)-10-(acetyloxy)-3,5-dihydroxy-4-[(1*S*,2*S*,6*S*,8*S*,9*R*,11*S*)-2-hydroxy-11-methyl-5,7,10-trioxatetracyclo[6.3.1.$0^{2,6}$.$0^{9,11}$]dodec-3-en-9-yl]-4-methyl-8-{[(2*E*)-2-methylbut-2-enoyl]oxy}octahydro-1*H*-furo[3',4':4,4a]naphtho[1,8-*bc*]furan-5,10a(8*H*)-dicarboxylate
**Epiafzelechin**	(2*R*,3*R*)-2-(4-hydroxyphenyl)-3,4-dihydro-2*H*-chromene-3,5,7-triol

Supplemental Figure 1

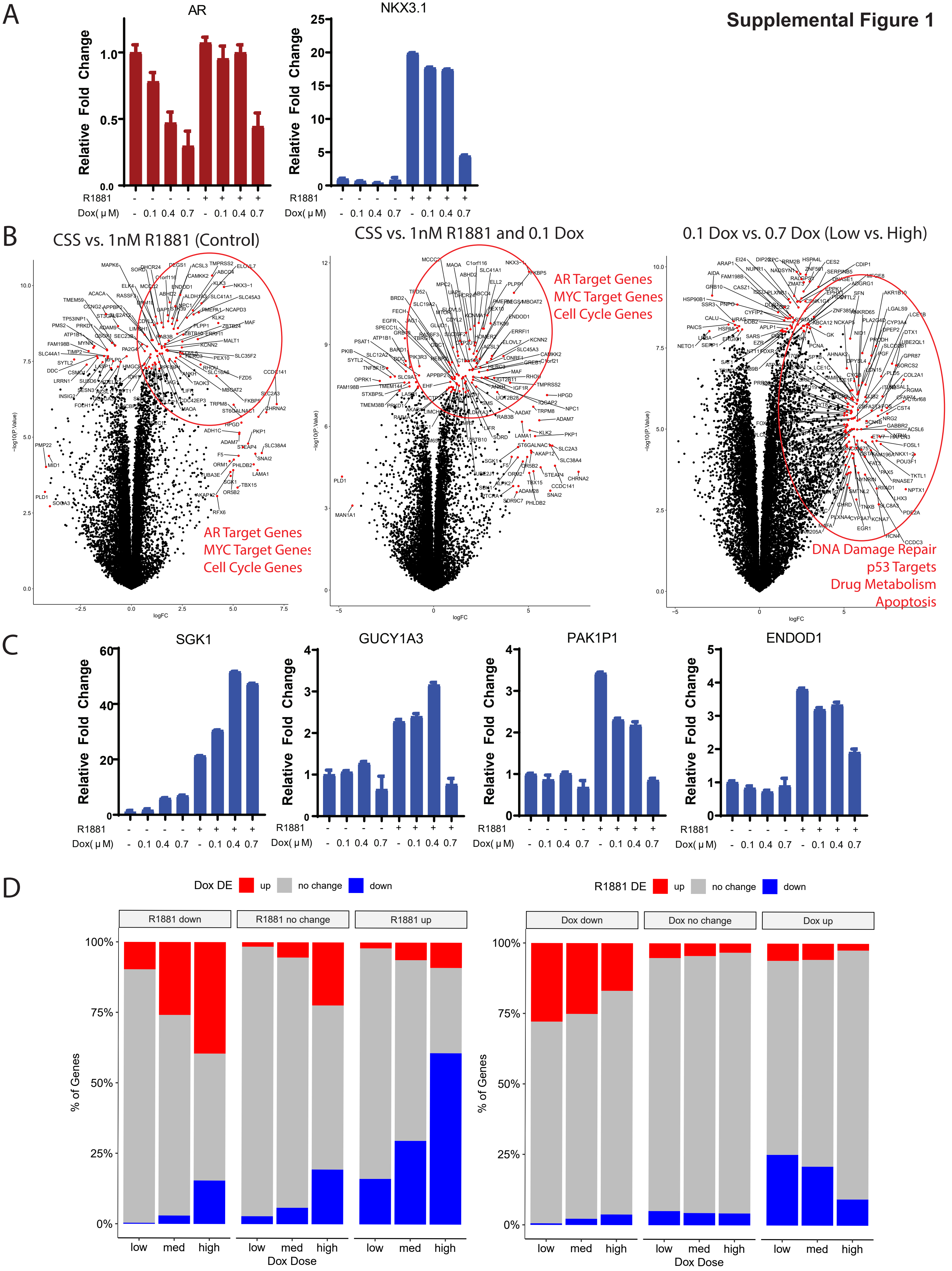

Supplemental Figure 1: Extended RNA-seq analysis. A) Normalized mRNA read-counts for AR and NKX3.1 from RNA-seq show both genes were relatively resistant to down-regulation by dox except at high doses. B) Differentially expressed genes from RNA-seq data were visualized by volcano plots, for conditions noted above each plot. Genes labeled and marked by red dots were most significant (P-value< 10^{-7}) with fold changes above the cut-off [Fold Change >abs(4)]. Top significantly relevant pathways identified by GSEA are highlighted through upregulated (red) genes encircled on plots. Panel on the left highlights the profound impact of androgen on gene expression that is only modestly changed by low dose dox (middle panel). The panel on the right highlights upregulation of cancer genes at high dose dox. C) Normalized mRNA read-counts for the sARE-like genes *SGK1*, *GUCY1A3*, *PAK1P1*, and *ENDOD1* from RNA-seq. These genes were identified as "sARE-like" for their resistance to downregulation by dox. D) Stacked barcharts of genes differentially expressed (DE) by dox and androgen. Percentage of genes either upregulated (red), not changed (gray), or downregulated (blue) for each of the low (0.1 µM), medium (med – 0.4 µM) or high (0.7 µM) doses of dox in the presence of androgen (1 nM R1881). Panels on the left illustrate the percentages of genes either decreased with androgen (R1881 down, left-most), not changed under androgen treatment (middle) or upregulated (R1881 up, right) that are either more downregulated with dox treatment (blue) or upregulated (red) in each condition. Panels on the right illustrate this similarly but relative to dox treatment, with the percentages of genes either decreased with dox treatment (left-most), not changed under any dox treatment (middle) or upregulated with dox (right) that are either more downregulated with androgen treatment (blue) or upregulated (red), respectively. Overall, increasing dox downregulates AR-induced genes and upregulates AR-repressed genes.

Supplemental Figure 2

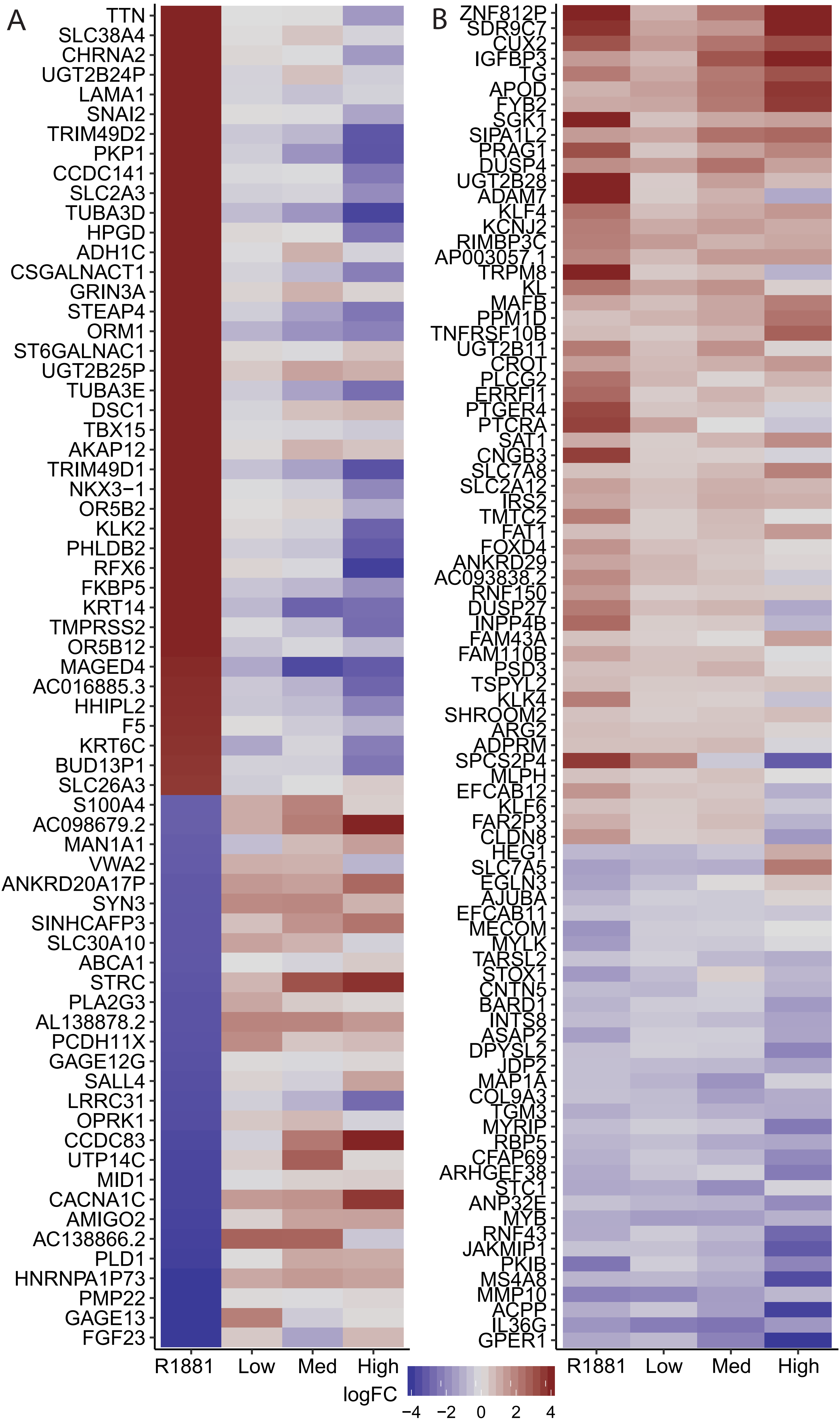

Supplemental Figure 2: Heat maps from RNA-seq data. A) The top genes that are androgen-induced (or androgen-repressed) and affected oppositely by dox are arrayed as a heat map. Genes that were upregulated with androgen (1 nM R1881) and also decreased with dox treatment are illustrated on top, with genes that were downregulated by androgen treatment and upregulated by dox shown on the bottom. B) Heat map of top dox-enhanced androgen-induced genes. Genes that were upregulated with androgen (1 nM R1881) and further induced with dox treatment are illustrated on top, with genes downregulated by androgen treatment and further downregulated by dox shown on the bottom.

Supplemental Figure 3: Top Significantly Enriched GSEA Pathways (p value >0.5): Control vs. 0.1 µM Dox (Low)

	pathway	pval	padj	ES	NES
1	DNA_Damage_Signaling_Pathway_DNA_Repair	8.556516e-05	0.009017028	−0.6499368	−1.980538
2	MYC_Targets_Up-Regulated_by MYC	8.612523e-05	0.009017028	−0.6649204	−2.000898
3	Cell_Cycle_Regulation_of Cell Cycle	8.907892e-05	0.009017028	−0.7346698	−2.040949
4	Molecular_Toxicology_PathwayFinder_Heat_Shock Res...	9.115770e-05	0.009017028	−0.7668951	−2.015073
5	DNA_Damage_Signaling_Pathway_ATM/ATR_Signaling	9.172629e-05	0.009017028	−0.8413587	−2.104021
6	DNA_Repair_Double-Strand_Break (DSB) Repair	9.254118e-05	0.009017028	−0.8774260	−2.109962
7	DNA_Repair_EpiTect_ChIP_qPCR_Array_Double-Strand...	9.254118e-05	0.009017028	−0.8774260	−2.109962
8	MYC_Targets_Metabolism	9.254118e-05	0.009017028	−0.8281700	−1.991516
9	Cancer_PathwayFinder_Cell_Cycle	9.665571e-05	0.009017028	−0.9092625	−1.869378
10	Cell_Cycle_M_Phase	9.674923e-05	0.009017028	−0.9545072	−2.001695
11	Cellular_Stress_Responses_Molecular_Chaperones	1.749475e-04	0.014822826	−0.6480313	−1.868818
12	JAK_or_STAT_Signaling_Pathway_Genes_Induced by S...	2.198527e-04	0.017075226	0.7419175	1.956996
13	Hypoxia_Signaling_Pathway_Responsive_Genes	2.576877e-04	0.018474225	−0.5784977	−1.748257
14	MYC_Targets_RNA_Processing & Binding Factors	2.899671e-04	0.019303527	−0.8703769	−1.789432
15	Apoptosis-2_TNFR_Domain	4.288624e-04	0.023608444	0.7886700	1.884481
16	DNA_Damage_Signaling_Pathway_EpiTect_ChIP_qPCR_...	4.306261e-04	0.023608444	−0.5829755	−1.744274
17	Hypoxia_Signaling_Pathway_Plus_Responsive_Genes	4.306261e-04	0.023608444	−0.5778001	−1.738733
18	Polycomb_and_Trithorax_Complexes_EpiTect_ChIP_qP...	4.671151e-04	0.024186182	−0.7906157	−1.855716
19	Immunotoxicity_Downregulated_in Immunotoxicity	7.473842e-04	0.036661160	−0.7790102	−1.828476
20	Cell_Cycle_EpiTect_ChIP_qPCR_Array_Regulation_of th...	8.028546e-04	0.037413024	−0.6491368	−1.795421
21	Notch_Signaling_Pathway_Plus_Notch_Target Genes	1.100473e-03	0.048840049	0.6796800	1.808841
22	Notch_Signaling_Pathway_Notch_Target Genes	1.324942e-03	0.056129363	0.6671384	1.801337

Top Significantly AR Enriched Pathways (p value >0.5): Control vs. 0.1 µM Dox (Low)

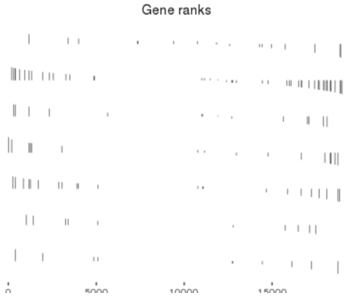

Pathway	NES	pval	padj
Prostate_Cancer_Androgen_Signaling	-1.20	2.3e-01	6.9e-01
Androgen_Receptor_Signaling_Targets_Up-Regulated_by Androgen Receptor	-1.15	2.3e-01	7.0e-01
Androgen_Receptor_Signaling_Targets_Transcriptional_Regulation	0.95	5.3e-01	9.0e-01
Androgen_Receptor_Signaling_Targets_Signal_Transduction	0.94	5.4e-01	9.0e-01
Androgen_Receptor_Signaling_Targets_Regulated_by Androgen Receptor	0.89	6.3e-01	9.6e-01
Androgen_Receptor_Signaling_Targets_Androgen_Receptor & Co-Transcription Factors	0.74	8.2e-01	1.0e+00
Androgen_Receptor_Signaling_Targets_Transporting_& Trafficking	-0.68	8.7e-01	1.0e+00

Supplemental Figure 4: Top Significantly Enriched GSEA Pathways (p value >0.5): Control vs. 0.4 μM Dox (Medium)

	pathway	pval	padj	ES	NES
1	Lipoprotein_Signaling_and_Cholesterol_Metabolism_C…	8.872327e-05	0.01496900	-0.5923973	-1.859418
2	DNA_Damage_Signaling_Pathway_DNA_Repair	8.884151e-05	0.01496900	-0.5985613	-1.889537
3	MYC_Targets_Up-Regulated_by MYC	8.922995e-05	0.01496900	-0.6662247	-2.074763
4	MYC_Targets_Metabolism	9.426848e-05	0.01496900	-0.7999138	-1.971324
5	Cancer_PathwayFinder_Cell_Cycle	9.592326e-05	0.01496900	-0.9207430	-1.930916
6	Cell_Cycle_M_Phase	9.636697e-05	0.01496900	-0.9012684	-1.932705
7	Cell_Cycle_Regulation_of Cell Cycle	1.823320e-04	0.01986677	-0.6996759	-2.013813
8	DNA_Damage_Signaling_Pathway_ATM/ATR_Signaling	1.877758e-04	0.01986677	-0.7199470	-1.846637
9	MYC_Targets_RNA_Processing & Binding Factors	1.918465e-04	0.01986677	-0.8852799	-1.856545
10	Cellular_Stress_Responses_Molecular_Chaperones	2.717391e-04	0.02366684	-0.6127170	-1.821895
11	Molecular_Toxicology_PathwayFinder_Heat_Shock Res…	2.793296e-04	0.02366684	-0.6950501	-1.883348
12	Hypoxia_Signaling_Pathway_Responsive_Genes	3.552713e-04	0.02759274	-0.5515963	-1.726723
13	Hypertension_Blood_Pressure Regulation	4.318722e-04	0.03096191	0.6797171	1.895965
14	Androgen_Receptor_Signaling_Targets_Up-Regulated…	6.368268e-04	0.04060244	-0.6070390	-1.775424
15	Insulin_Resistance_Metabolic_Pathways	6.534727e-04	0.04060244	-0.6803631	-1.806269
16	Hypoxia_Signaling_Pathway_Plus_Responsive_Genes	8.030695e-04	0.04677880	-0.5442062	-1.694772
17	Ubiquitin_Ligases_Single_RING-Finger	9.080296e-04	0.04978139	0.3807362	1.427522
18	Glucose_Metabolism_Glucose_Metabolism	1.152074e-03	0.05965182	-0.5220488	-1.642588
19	Amino_Acid_Metabolism_II_Histidine_Metabolism	1.252217e-03	0.06142456	0.8019575	1.779327
20	Apoptosis-2_TNFR_Domain	1.581945e-03	0.06845835	0.7408040	1.796595
21	DNA_Damage_Signaling_Pathway_EpiTect_ChIP_qPCR_…	1.609442e-03	0.06845835	-0.5382308	-1.664323
22	Telomeres_and_Telomerase_Other_Genes Associated …	1.615970e-03	0.06845835	-0.7576430	-1.800528

Top Significantly AR Enriched Pathways (p value >0.5): Control vs. 0.4 μM Dox (Med)

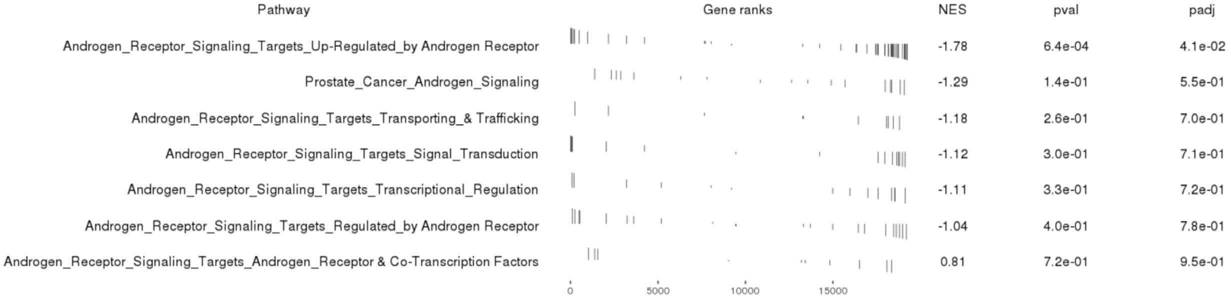

Supplemental Figure 5: Top Significantly Enriched GSEA Pathways (p value >0.5): Control vs. 0.7 µM Dox (High)

	pathway	pval	padj	ES	NES
1	Drug_Metabolism-_Phase_I_Enzymes_Cytochrome_P450	9.409108e-05	0.01445791	0.5854407	1.924431
2	Cell_Cycle_M_Phase	1.037129e-04	0.01445791	-0.8365625	-2.014365
3	DNA_Damage_Signaling_Pathway_ATM/ATR_Signaling	1.050862e-04	0.01445791	-0.6964408	-2.056522
4	Cell_Cycle_Regulation_of Cell Cycle	1.066780e-04	0.01445791	-0.6404547	-2.142938
5	Androgen_Receptor_Signaling_Targets_Up-Regulated…	1.075500e-04	0.01445791	-0.6737983	-2.291529
6	MYC_Targets_Up-Regulated_by MYC	1.081549e-04	0.01445791	-0.6369902	-2.331723
7	DNA_Damage_Signaling_Pathway_DNA_Repair	1.085894e-04	0.01445791	-0.5408499	-2.009028
8	Apoptosis_Induction_of Apoptosis	1.871783e-04	0.01526668	0.5640153	1.903903
9	Hematopoiesis_Cell_Differentiation	1.880229e-04	0.01526668	0.5894780	1.921092
10	Cancer_PathwayFinder_Cell_Cycle	2.088119e-04	0.01526668	-0.8528931	-1.994508
11	MYC_Targets_RNA_Processing & Binding Factors	2.088119e-04	0.01526668	-0.8557032	-2.001080
12	Mitochondria_Inner_Membrane Translocation	2.100620e-04	0.01526668	-0.7646558	-2.006847
13	Cell_Cycle_EpiTect_ChIP_qPCR_Array_Regulation_of th…	2.129472e-04	0.01526668	-0.5803208	-1.932820
14	Molecular_Toxicology_PathwayFinder_Apoptosis	2.841986e-04	0.01891950	0.6440044	1.984721
15	DNA_Damage_Signaling_Pathway_EpiTect_ChIP_qPCR_…	3.257329e-04	0.02023887	-0.4940427	-1.790752
16	p53_Signaling_Pathway_Target_Genes	4.721881e-04	0.02750496	0.5891569	1.877530
17	MYC_Targets_Metabolism	5.220297e-04	0.02861951	-0.6820345	-1.927142
18	Toll-Like_Receptor_Signaling_Pathway_Downstream_P…	6.546951e-04	0.03389866	0.5368492	1.805159
19	MYC_Targets_Protein_Synthesis, Degradation & Turno…	8.342023e-04	0.03729671	-0.7087676	-1.922893
20	Transplant_Rejection_Acute_Rejection - Innate Immu…	8.394739e-04	0.03729671	0.5208795	1.767293
21	Angiogenic_Growth_Factors_Angiogenic_Growth Fact…	8.408072e-04	0.03729671	0.5316221	1.781981
22	Cell_Death_PathwayFinder_Autophagy	9.460738e-04	0.03729671	0.5870294	1.822457
[illegible]	[illegible]	[illegible]	[illegible]	[illegible]	[illegible]

Top Significantly AR Enriched Pathways (p value >0.5): Control vs. 0.7 µM Dox (High)

Pathway	NES	pval	padj
Androgen_Receptor_Signaling_Targets_Up-Regulated_by Androgen Receptor	-2.29	1.1e-04	1.4e-02
Androgen_Receptor_Signaling_Targets_Transcriptional_Regulation	-1.67	1.3e-02	1.5e-01
Androgen_Receptor_Signaling_Targets_Transporting_& Trafficking	-1.61	2.5e-02	1.9e-01
Androgen_Receptor_Signaling_Targets_Signal_Transduction	-1.55	3.2e-02	2.1e-01
Androgen_Receptor_Signaling_Targets_Regulated_by Androgen Receptor	-1.50	3.8e-02	2.2e-01
Prostate_Cancer_Androgen_Signaling	-0.79	7.8e-01	8.9e-01
Androgen_Receptor_Signaling_Targets_Androgen_Receptor & Co-Transcription Factors	0.74	8.2e-01	9.2e-01

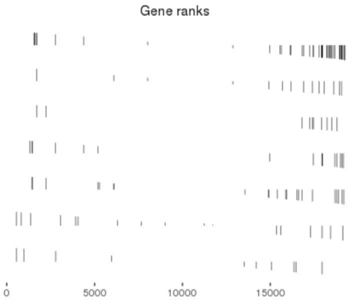

Supplemental Figure 6: Top Significantly Enriched GSEA Pathways (p value > 0.5): 0.1 μM Dox (Low) vs. 0.7 μM Dox (High)

	pathway	pval	padj	ES	NES
1	Transplant_Rejection_Acute_Rejection – Innate Immu…	8.974244e-05	0.02674779	0.6291397	2.028287
2	p53_Signaling_Pathway_Target_Genes	9.084302e-05	0.02674779	0.6548209	1.989596
3	Androgen_Receptor_Signaling_Targets_Up-Regulated…	1.126761e-04	0.02674779	−0.7466532	−2.457246
4	MYC_Targets_Up-Regulated_by MYC	1.147974e-04	0.02674779	−0.5954591	−2.095090
5	Apoptosis_Induction_of Apoptosis	1.790991e-04	0.02792007	0.5941934	1.909973
6	Angiogenic_Growth_Factors_Angiogenic_Growth Fact…	1.797430e-04	0.02792007	0.6348131	2.024458
7	Hematopoiesis_Cell_Differentiation	2.716899e-04	0.02819884	0.6150972	1.907070
8	Molecular_Toxicology_PathwayFinder_Apoptosis	2.723064e-04	0.02819884	0.6607141	1.943439
9	TGFbeta_Signaling_Targets_Signal_Transduction	2.723064e-04	0.02819884	0.6583300	1.936426
10	Drug_Metabolism-_Phase_I_Enzymes_Cytochrome_P450	3.611412e-04	0.03181050	0.6015531	1.884196
11	Tight_Junctions_Cytoskeleton_Regulators	3.754458e-04	0.03181050	0.7853747	1.903676
12	Notch_Signaling_Targets_Developmental_ Processes	5.329070e-04	0.04138911	0.5451803	1.799618

Top Significantly AR Enriched Pathways (p value>0.5): 0.1 μM Dox (Low) vs. 0.7 μM Dox (High)

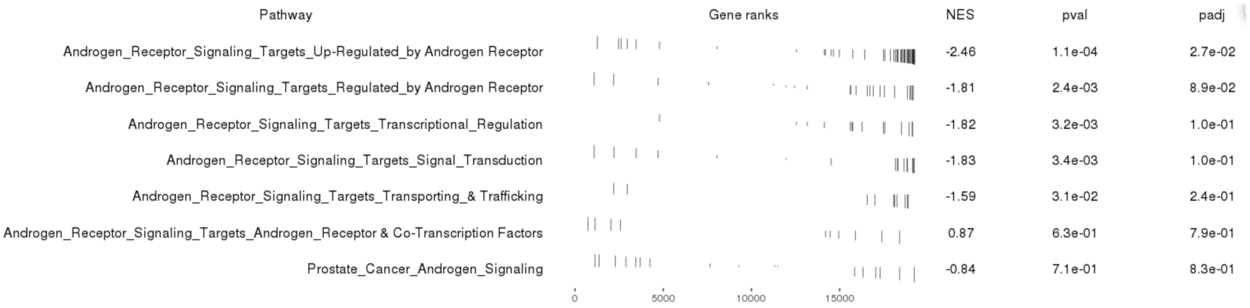

Supplemental Figure 7:
MSigDB Pathway Heatmap

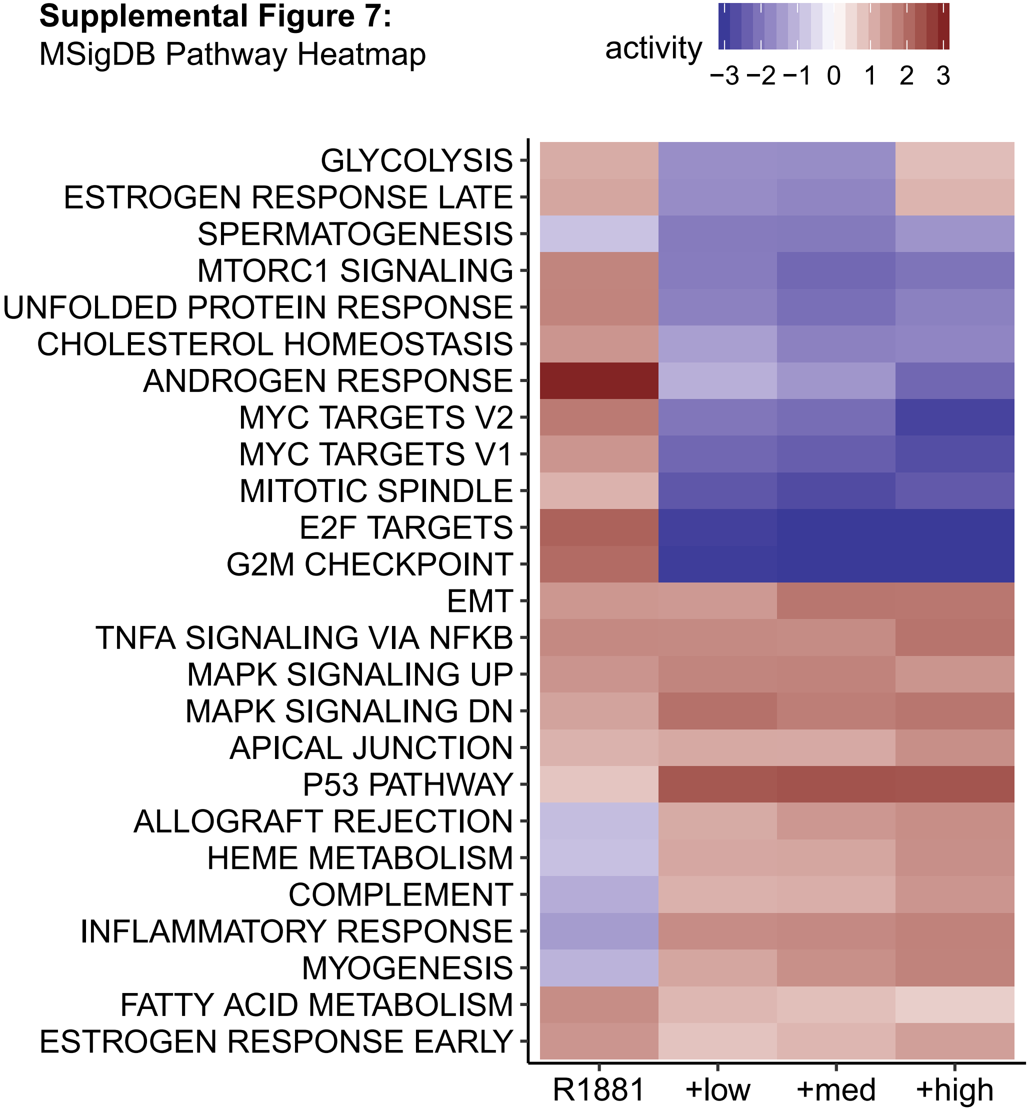

Supplemental Figure 7: Heat map from RNA-seq Pathways. Heatmap illustrating the impact of dox on the activity of “Hallmark” MSigDB pathways from RNA-seq of LNCaP cells starved for 24 hours in charcoal stripped serum and treated with androgen alone (R1881 – 1 nM) or each of the low (0.1 µM), medium (med – 0.4 µM) or high (0.7 µM) doses of doxorubicin in the presence of androgen (1 nM R1881).

Supplemental Figure 8

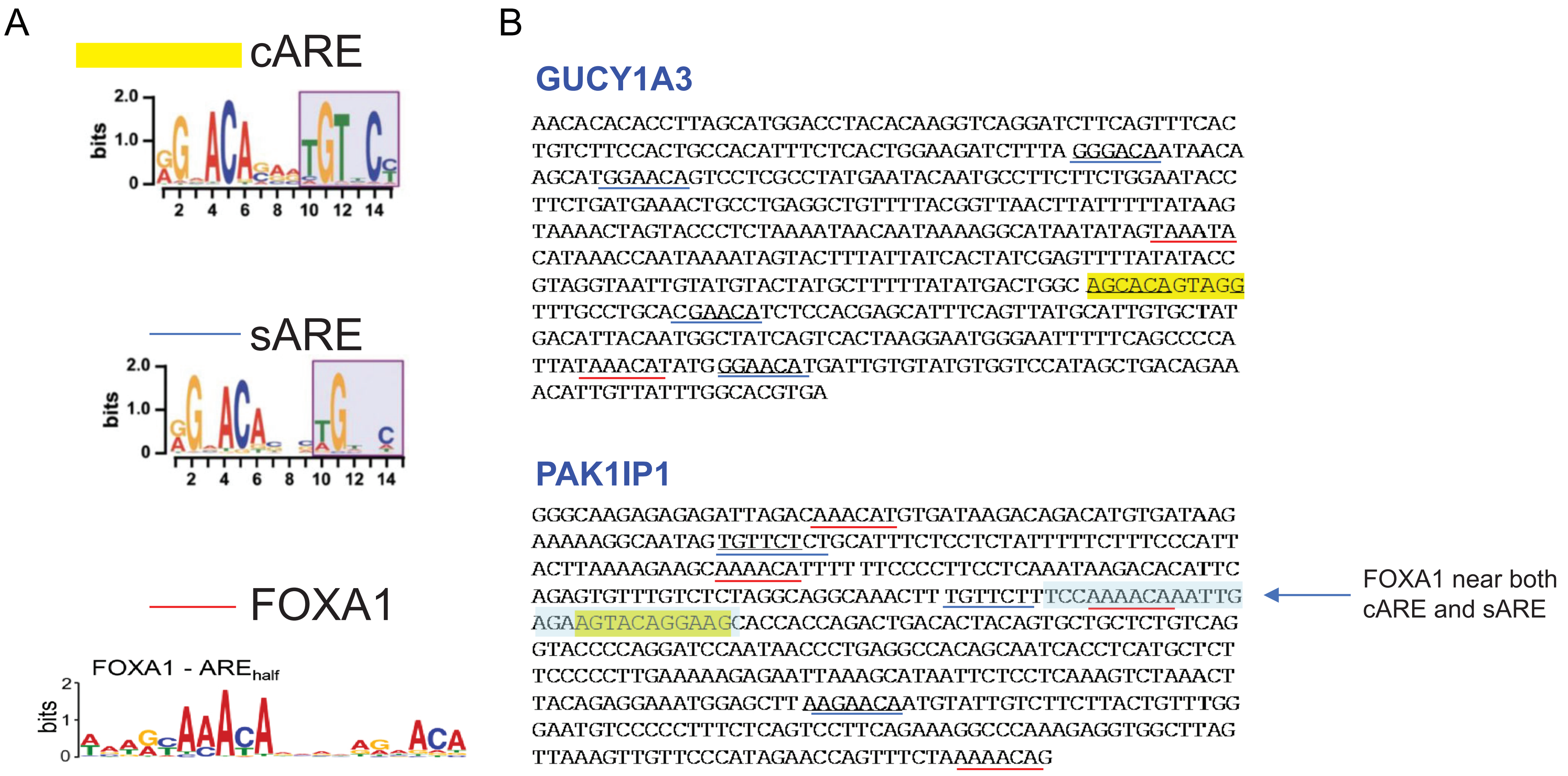

Supplemental Figure 8: Identification of response element motifs within the ChIP-seq peaks of candidate sARE-like genes GUCY1A3 and PAK1IP1. A) Key of the motifs identified for each of the androgen response elements: cARE (highlighted in yellow), sARE (underlined in blue), and the AR cofactor FOXA1 (underlined in red). B) DNA sequence corresponding to the ChIP-seq peaks identified in Figure 5 in the promoters of GUCY1A3 (top) and PAK1IP1 (bottom). The response elements are highlighted in yellow for cAREs, underlined in blue for sAREs, or red for FOXA1. A unique site with all three response elements is highlighted in blue in the PAK1IP1 peak.

Supplemental Figure 9

A: Relative Peak Distribution of AR Peaks in Chromatin

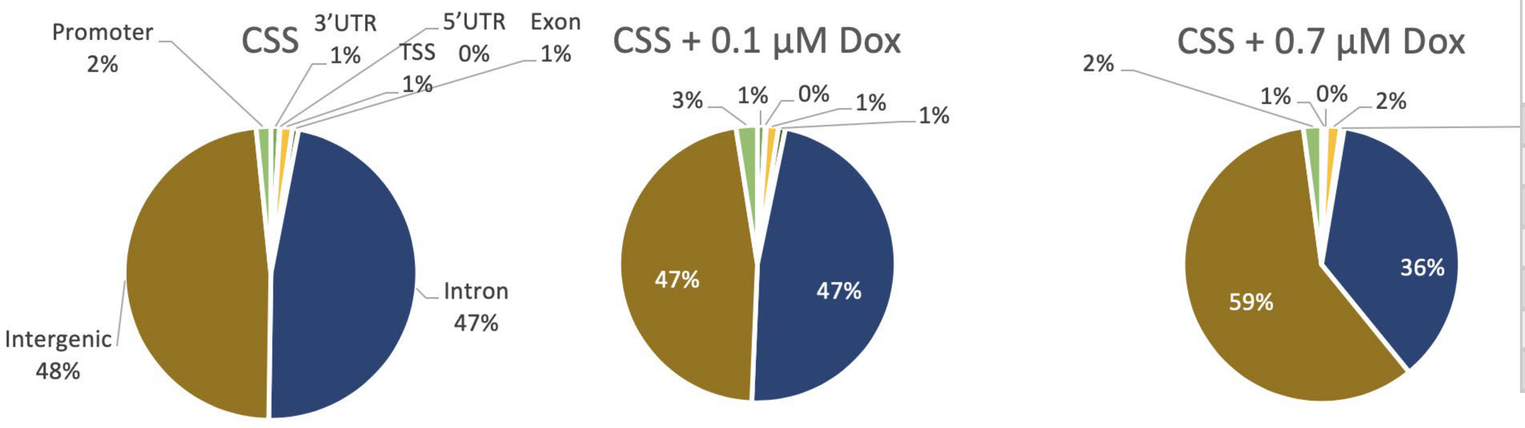

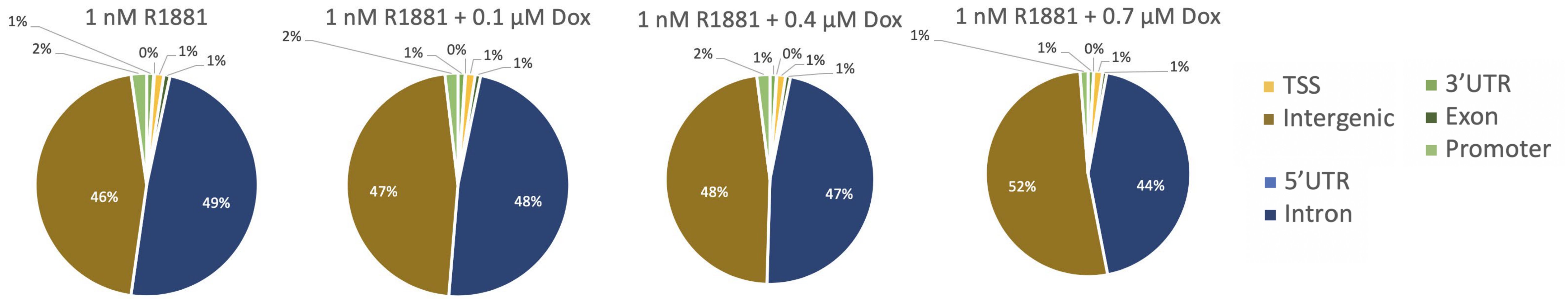

B: Table of Percentage of Peaks in Genomic Region

	CSS	CSS + 0.1 µM Dox	CSS + 0.7 µM Dox	1 nM R1881	1 nM R1881 + 0.1 µM Dox	1 nM R1881 + 0.4 µM Dox	1 nM R1881 + 0.7 µM Dox
3'UTR	0.9	0.9	0.5	1.0	1.0	1.0	0.9
5'UTR	0.1	0.1	0.0	0.1	0.1	0.1	0.0
TSS	1.4	1.4	1.6	1.3	1.4	1.3	1.4
Exon	0.7	0.8	0.4	0.9	0.8	0.8	0.7
Intron	47.2	47.4	36.5	48.9	48.0	47.3	44.0
Intergenic	48.1	46.9	58.8	45.5	46.8	47.5	51.8
Promoter	1.6	2.5	2.1	2.2	1.9	2.0	1.3

Supplemental Figure 9: Relative distribution of AR-peaks in chromatin under ChIP-seq conditions. A) Pie charts showing the percentage of ChIP-seq peaks identified near the transcriptional start site (TSS – yellow), in the 3' untranslated region (3' UTR - green), in an exon (dark green), the 5' untranslated region (5' UTR – light blue), in introns (dark blue), or the promoters of genes (light green). Intergenic peaks not near genes are shown in brown. B) Table of the percentages of the peaks in each of described genomic regions. No significant changes in AR peak distribution relative to gene structure are caused by dox treatment at lower doses.
